# Aluminum-based metal–organic framework nanoparticles as pulmonary vaccine adjuvants

**DOI:** 10.1186/s12951-023-01782-w

**Published:** 2023-02-03

**Authors:** Zachary S. Stillman, Gerald E. Decker, Michael R. Dworzak, Eric D. Bloch, Catherine A. Fromen

**Affiliations:** 1 Department of Chemical and Biomolecular Engineering, University of Delaware, 150 Academy St., Newark, DE 19716 USA; 2 Department of Chemistry and Biochemistry, University of Delaware, 150 Academy St., Newark, DE 19716 USA

**Keywords:** Metal–organic framework, Pulmonary, Vaccine, Adjuvant

## Abstract

The adoption of pulmonary vaccines to advantageously provide superior local mucosal protection against aerosolized pathogens has been faced with numerous logistical and practical challenges. One of these persistent challenges is the lack of effective vaccine adjuvants that could be well tolerated through the inhaled route of administration. Despite its widespread use as a vaccine adjuvant, aluminum salts (alum) are not well tolerated in the lung. To address this issue, we evaluated the use of porous aluminum (Al)-based metal–organic framework (MOF) nanoparticles (NPs) as inhalable adjuvants. We evaluate a suite of Al-based MOF NPs alongside alum including DUT-4, DUT-5, MIL-53 (Al), and MIL-101﻿-NH_2_ (Al). As synthesized, MOF NPs ranged between ~ 200 nm and 1 µm in diameter, with the larger diameter MOFs matching those of commercial alum. In vitro examination of co-stimulatory markers revealed that the Al-based MOF NPs activated antigen presenting cells more effectively than alum. Similar results were found during in vivo immunizations utilizing ovalbumin (OVA) as a model antigen, resulting in robust mucosal humoral responses for all Al MOFs tested. In particular, DUT-5 was able to elicit mucosal OVA-specific IgA antibodies that were significantly higher than the other MOFs or alum dosed at the same NP mass. DUT-5 also was uniquely able to generate detectable IgG2a titers, indicative of a cellular immune response and also had superior performance relative to alum at equivalent Al dosed in a reduced dosage vaccination study. All MOF NPs tested were generally well-tolerated in the lung, with only acute levels of cellular infiltrates detected and no Al accumulation; Al content was largely cleared from the lung and other organs at 28 days despite the two-dose regime. Furthermore, all MOF NPs exhibited mass median aerodynamic diameters (MMADs) of ~ 1.5–2.5 µm when dispersed from a generic dry powder inhaler, ideal for efficient lung deposition. While further work is needed, these results demonstrate the great potential for use of Al-based MOFs for pulmonary vaccination as novel inhalable adjuvants.

**Graphical Abstract:**

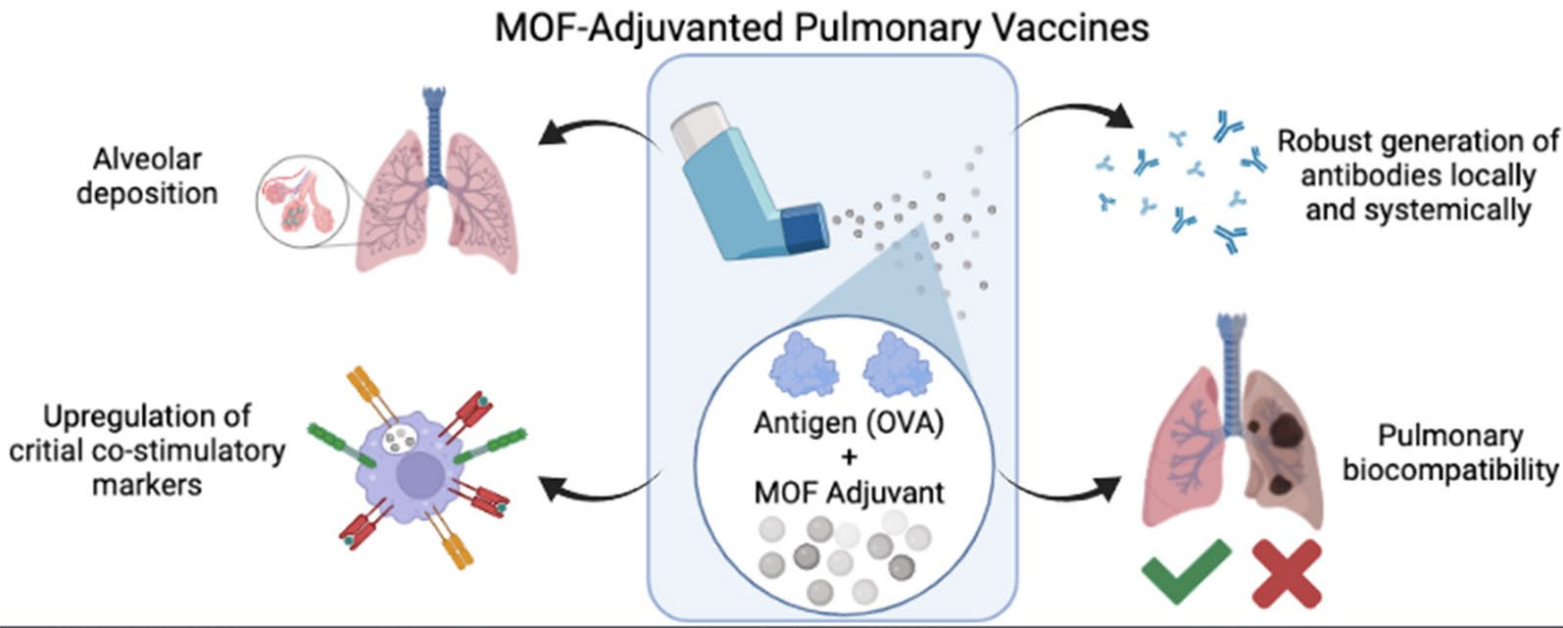

**Supplementary Information:**

The online version contains supplementary material available at 10.1186/s12951-023-01782-w.

## Introduction

Inhalable vaccines offer significant opportunity to elicit local mucosal immune responses needed to engender the most robust protection against inhalable pathogens, while also providing advantages for needle-free administration and mitigating cold-chain storage issues [1, 2]. As a critical example, currently licensed COVID-19 vaccines employ intramuscular immunizations that fail to elicit mucosal responses [3], pointing to the need for inhalable vaccination strategies [4]. Some of the critical challenges for pulmonary vaccination include: (1) limited and sometimes ineffective delivery devices, (2) variations in particle sizes leading to inconsistent delivery of vaccine cargo, and (3) lack of safe and effective vaccine adjuvants [5, 6]. Indeed, development of novel inhalable adjuvants, *i.e.* immune stimulating agents, that are well tolerated in the airway remain a critical need for both pulmonary and nasal immunization strategies [1, 2, 7].

The search for safe and effective adjuvants broadly has been a difficulty since their inception, with only a small handful of adjuvants reaching approval for human use. Among the recently approved adjuvants are oil-in-water emulsions such as MF59 or Freund’s adjuvant, immunostimulating complexes such as ISCOM and ISCOMATRIX, and compounds such as monophosphoryl lipid A (MPL A—formulated into AS04, AS02A, etc.) and oligodeoxynucleotides (CpG-1018) that belong to a class of conserved pathogen-associated molecular patterns (PAMP) that signal through toll-like receptors (TLRs) [8]. Compared to these newer formulations, alum remains the most commonly used vaccine adjuvant, with widespread use over the last ~ 80 years [9]. Alum is a formulation of aluminum salts generally composed of aluminum oxyhydroxide, aluminum phosphate, and/or aluminum potassium sulfate and has a track record of safety and effectiveness in vaccines [10, 11]. Its mechanism of action remains an on-going point of investigation [9, 12]; alum is believed to cause an enhanced immune response by activating the NLRP3 inflammasome and also causes a “depot effect” in which there is sustained release of the antigen over time at the site of injection while simultaneously recruiting leukocytes to the site, aiding in their education and enhancement of the immune response [13, 14]. Alum is also uniquely able to generate a strong T helper 2 (Th2) type response, leading to high levels of antigen-specific antibody generation that confer critical protection to the host [11]. Alum has been used to adjuvant a wide range of vaccine platforms, including attenuated, inactivated, protein, and subunit vaccines, providing a cost-effective, versatile, and robust adjuvant approach [9, 15]. Despite these various benefits and its relatively long historical use, alum has yet to be translated to the inhaled route of administration because of pervasive safety concerns of causing or worsening inflammation in the lungs [16, 17].

Inspired by the chemical similarity to alum, we sought to explore aluminum (Al)-based metal–organic framework (MOF) nanoparticles (NPs) as pulmonary vaccine adjuvants. MOFs are a versatile class of hybrid materials composed of metal-based clusters connected in three dimensions by organic linkers with highly tailorable properties (i.e., pore size, cargo loading, geometric size, etc.) and inherent porosity that make this class of materials potentially advantageous for biomedical and aerosol applications [18, 19]. The chemical similarity of MOF Al-based metal clusters to alum is hypothesized to provide inherent immunogenicity, while limiting the overall Al dosed to mitigate adverse responses to the lung. In this work, we synthesize a series of Al-containing MOF nanomaterials and evaluate their ability to (1) provide advantageous deposition based on aerosol size, (2) activate antigen presenting cells (APCs) in vitro and in vivo, (3) generate local and systemic antibodies following pulmonary vaccination in a murine model, (4) recruit immune cells to the lungs without causing undue inflammation, and (5) avoid accumulation in the airspace following direct lung delivery. Compared to alum, our results demonstrate that MOFs exhibit significant advantages as vaccine adjuvants across a range of relevant attributes. Through this assessment, we demonstrate the feasibility of using Al-MOFs as novel aerosol adjuvants to increase the immunogenicity of a model protein antigen and also introduce these materials as potential vaccine adjuvants more broadly.

## Experimental section

### Materials

Alum was obtained from G Biosciences and was washed three times with sterile water prior to use in any in vitro or in vivo studies. Lyophilized Endofit ovalbumin, a low endotoxin formulation, was obtained from Invitrogen and was kept sterile and only resuspended in physiological, endotoxin-free water (Invitrogen) prior to use in in vitro or in vivo studies. For all in vitro studies, dilutions and formulations were performed using sterile Dulbecco’s Modified Eagle Medium (DMEM) (Corning) supplemented with 10% fetal bovine serum (FBS, Gibco) and 1% penicillin–streptomycin or sterile phosphate buffered saline (PBS, Alfa Aesar). All other reagents were obtained from Fisher Scientific unless otherwise noted within subsections below.

### Synthesis of Al-based metal–organic framework (MOF) nanoparticles (NPs)

#### DUT-4 synthesis

2,6-Naphthalene dicarboxylic acid (0.26 g, 1.2 mmol) and DMF (30 mL) were combined and sonicated until homogeneous. Al(NO_3_)_3_·9H_2_O (0.52 g, 1.4 mmol) was then added to the solution and the mixture was once again sonicated until homogenous. The solution was then transferred to a Teflon-lined steel autoclave and heated to 120 °C for 24 h. After being cooled to room temperature, the product was obtained by centrifugation, where the solid was washed three times with fresh DMF and three times with methanol [20]. The sample was activated at 190 °C under flowing N_2_ for a minimum of 24 h before gas adsorption or biological studies were conducted.

#### DUT-5 synthesis

4,4′-Biphenyl dicarboxylic acid (0.26 g, 1.2 mmol) and Al(NO_3_)_3·_9H_2_O (0.52 g, 1.4 mmol) and DMF (30 mL) were combined in a beaker and sonicated until homogeneous. The solution was then transferred to a Teflon-lined steel autoclave heated at 120 °C for 24 h. Then the autoclave was cooled down to room temperature and the solid was obtained by centrifugation and then washed with DMF three times and three times with methanol [20]. The sample was activated at 190 °C under flowing N_2_ for a minimum of 24 h before gas adsorption or biological studies were conducted.

#### MIL-53 (Al) synthesis

Al(NO_3_)_3_·9H_2_O (3.9 g, 18.3 mmol) and terephthalic acid (0.96 g, 5.8 mmol) were added to a beaker and suspended in H_2_O (15 mL). The mixture was sonicated for 10 min before being transferred to a Teflon-lined steel autoclave and heated at 220 °C for 72 h. After allowing the vessel to cool to room temperature, the mixture was transferred to a scintillation vial and the product was isolated via centrifugation. The sample was washed thrice with DMF, thrice with methanol and activated at 100 °C under flowing N_2_ for a minimum of 24 h before gas adsorption or biological studies were conducted [21].

#### MIL-101-NH_2_ (Al) synthesis

AlCl_3_·6H_2_O (0.51 g, 2.1 mmol) and 2-amino terephthalic acid (0.56 g, 3.4 mmol) were added to a beaker and solvated in DMF (30 mL). The solution was then transferred to a Teflon-lined steel autoclave and heated at 130 °C for 72 h. The product was obtained by centrifugation and washed three times with acetone. The solid was then suspended in methanol and heated under reflux for 18 h before being cooled to room temperature and collected again via centrifugation. The sample was activated at 100 °C under flowing N_2_ for a minimum of 24 h before gas adsorption or biological studies were conducted [22].

### Dynamic light scattering (DLS)

DLS was performed using a Malvern Zetasizer Nano ZS for all Al-based MOFs. Alum and the as synthesized MOFs were washed in water three times prior to DLS measurement to remove any adsorbed ligands or stabilizers. The washing procedure involved centrifugation at 18.2 K RCF for 5 min followed by removal of the supernatant and redispersion into water using sonication. The NP sample concentrations were then adjusted to 0.1 mg/mL following concentration determination via thermogravimetric analysis (TGA). DLS measurements were then performed to determine the hydrodynamic diameters (D_h_) for each sample. Reported measurements for each NP were averages taken from three samples synthesized at the same synthetic conditions.

Zeta potential measurements were performed using a Malvern Zetasizer Nano ZS following additional washing steps into 0.1 × PBS. The washing procedure involved centrifugation at 18.2 K RCF for 5 min followed by removal of the supernatant and redispersion into water solution using sonication two times and then into 0.1 × PBS once. The NP sample concentrations were then adjusted to 1 mg/mL following concentration determination via TGA. Zeta potential measurements were then performed to determine the surface charge of the NPs. Reported measurements for each NP were averages taken from three samples synthesized at the same synthetic conditions.

### Scanning electron microscopy (SEM)

Particle samples were prepared for SEM by pipetting 1 µL of sample at 1 mg/mL onto a glass slide and evaporating solvent overnight. SEM was then performed using a JSM F7400 scanning electron microscope after each of the samples was sputter coated with gold/palladium (thickness of ~ 5 nm) using a Denton Desk IV sputter coater. Imaging was performed using the secondary electron detection imaging mode. The geometric diameters (D_g_) of the NPs were determined using the ImageJ program to manually determine the diameters of at least 50 NPs.

### Thermogravimetric analysis (TGA) for concentration determination

NP concentrations in their dispersions in water or 0.1x PBS were determined via TGA using a TA Instruments TGA 550. A known volume of the dispersion was heated to 110 °C to evaporate all solvent present. The temperature was then held constant for 15 min to ensure complete evaporation and removal of solvent from the pores of the NPs. The remaining mass was deemed to be only NP and used to determine the mass concentration in solution.

### Next generation impactor (NGI) sizing

The aerodynamic diameters of the five respective Al-based NPs were measured using cascade impaction in a Copley Next Generation Impactor (NGI). A PlastiApe Monodose dry powder inhaler (DPI) was utilized to aerosolize the NPs. Dry powders of each NP sample were generated following lyophilization of aqueous particle dispersions. Approximately 5 mg of dry powder was loaded into a size 3 gelatin capsule and dispersed from the DPI under NGI actuation. For both dispersion devices, the NGI was operated at 60 L/min for 80 s to ensure sufficient mass accumulation on the impactor stages. Particles were collected from each stage by scraping and dispersing into water. Mass deposition was quantified using TGA and absorbance readings on a BioTek Cytation 5 Multimode Imager. The absorbance assay utilized the absorbance of the respective NPs (maximum absorbance was generally at ~ 290 nm). From the deposition profiles on each plate, the mass median aerodynamic diameter (MMAD) and geometric standard deviation (GSD) were determined using particle size cutoffs for an NGI operating at 60 L/min [23].

### In vitro cell assays: viability

The RAW264.7 (ATCC TIB-71) murine macrophage cell line was cultured according to ATCC guidelines. All experiments were performed with cell lines not exceeding a passage number of 10. For in vitro cell viability assessment, RAW264.7 cells were seeded in 96-well plates 12 h prior to treatment to allow for adherence. Immediately prior to NP treatment, the respective NPs were washed 3 times with sterile, endotoxin-free water and then resuspended in sterile DMEM (Corning) supplemented with 10% fetal bovine serum (Gibco) and 1% Penicillin–Streptomycin (GE Healthcare HyClone™). 24 h following treatment, cell viability was assessed using CellTiter-Glo^®^ 2.0 Cell Viability Assay (Promega) according to manufacturer’s guidelines. Luminescence was recorded using BioTek Cytation 5 Multimode Imager and cell viability was calculated from luminescence data by normalizing to the untreated control. Details for dosage amounts and groups for this study and others are summarized in Table 1.

**Table 1:** Summary table for dosage timelines, amounts/concentrations, groups, and time points for in vitro and in vivo studies referenced throughout this work using the Al-based NPs

Study	Dosage time point(s)	Dosage amounts or concentrations (per time point)	Dosage groups w/replicates (N)	Time point (end)
In vitro studies	0 h	100 µg/mL NPs	UT (3), Al-based NPs (3)	24 h
In vivo antibody (equal mass)	0 days, 14 days	100 µg/mouse NPs + 25 µg/mouse OVA	PBS (2), OVA only (4), alum + OVA, (4), Al-based MOFs + OVA (5)	28 days
Dose sparing in vivo antibody (equal Al)	0 days, 14 days	25 (alum) or 50 (DUT-5) µg/mouse + 25 µg/mouse OVA	PBS (3), alum + OVA (5), DUT-5 + OVA (5)	28 days
In vivo surface marker expression (equal mass)	0 h	100 µg/mouse NPs + 25 µg/mouse OVA	PBS (2), OVA only (4), alum + OVA, (4), Al-based MOFs + OVA (5)	24 h
In vivo biodistribution (equal mass)	A: 0 days B: 0 days, 14 days	A, B: 100 µg/mouse NPs + 25 µg/mouse OVA	A, B: PBS (2), OVA only (4), alum + OVA, (4), Al-based MOFs + OVA (5)	A: 24 h B: 28 days

### In vitro cell assays: uptake and fluorescent imaging

The five respective Al-based NPs were loaded with fluorescein isothiocyanate (FITC) (Millipore Sigma) at an incubation ratio of 1:1 in water at 37 °C and 1000 rpm (3.3 rcf) for 24 h. RAW264.7 cells were seeded in a 96 well place and allowed adhere for 12 h before NP treatment. 24 h following treatment, cells were washed with PBS and imaged on the BioTek Cytation 5 Multimode Imager. To differentiate between internalization and surface binding of Al-based NPs, RAW264.7 cells were washed once with PBS to remove free NPs and then Trypan Blue dye (Gibco) was added at a final concentration of 2 mg/mL to quench any surface associated FITC signal. Another subset of Al-based NP-treated RAW cells were washed with FACS buffer (96% PBS, 4% FBS by volume) and then analyzed using ACEA NovoCyte Flow Cytometer to detect levels of uptake based on median fluorescent intensity (MFI) on the FITC channel of the flow cytometer with, baseline MFI set based on untreated cells. Details for dosage amounts and groups for this study and others are summarized in Table 1.

### Animals

All studies involving animals were performed in accordance with National Institutes of Health (NIH) guidelines for the care and use of laboratory animals and approved by the Institutional Animal Care and Use Committee (IACUC) at the University of Delaware. Female C57BL/6 J (Jackson Laboratories) were housed in a pathogen-free facility at the University of Delaware and given unrestricted access to chow and water. Mice 4–8 weeks of age were used in vaccination, APC activation, histology, and biodistribution studies and all doses given via orotracheal instillation. For these studies, initial dosages were all given to mice at 4 weeks of age and comparable weight with details for dosage in each of the following subsections. For each of the respective studies, the desired dosage of particles detailed in the following sections were delivered in 50 µL doses to the mice, which had been anesthetized with isoflurane [24].

### In vivo murine alveolar macrophage activation studies

To assess acute activation of alveolar macrophages, key APCs present in the lungs, 50 µL volumes of Al-based NPs dispersions with ovalbumin (OVA) and controls (PBS only or OVA only) in PBS were administered to 4-week-old female C57BL/6 J mice via orotracheal instillation. 100 µg NPs (determined via TGA) and 25 µg OVA were administered per mouse for each of the respective treatments (except for PBS only and OVA only controls). 24 h following dosage, mice were euthanized and bronchoalveolar lavage (BAL) was performed to collect BAL fluid (BALF) by cannulating the trachea and flushing the lungs with two sequential washes, 1 mL each, of PBS. A summary of information regarding the dosage amounts and groups for this study are summarized in Table 1. The collected BALF was centrifuged at 500 RCF for 5 min. The cell pellet was washed twice with PBS supplemented with 4% fetal bovine serum and red blood cells lysed using RBC lysis buffer (Alfa Aesar). BALF cells were then stained for 30 min with the following antibodies: CD45 (FITC, clone: 30-F11), Ly6G (APC, clone: RB8-8C5), CD86 (AlexaFluor700), I-A/I-E (Brilliant Violet 785, clone: M5/114.15.2) (all from BioLegend), Siglec-F (PE-Cy7, clone: E50 2440) (BD Biosciences), and CD40 (SuperBright 600, clone: 1C10) (Invitrogen). Cells were then analyzed using ACEA NovoCyte Flow Cytometer for isolation of alveolar macrophage and neutrophil populations; median fluorescent intensity (MFI) was recorded via flow cytometry as a measure of surface marker expression.

### In vivo murine vaccination studies

To assess vaccination effectiveness, 4-week-old female C57BL/6 J mice were immunized via prime/boost dosages of 100 µg NPs alongside 25 µg OVA via orotracheal instillation for the equivalent mass experiments, with concentrations determined via TGA. The included groups, noted in Table 1 for the various in vivo experiments, generally included mice dosed with only PBS, only OVA in PBS, alum plus OVA, DUT-4 plus OVA, DUT-5 plus OVA, MIL-53 (Al) plus OVA, and MIL-101-NH_2_ (Al) plus OVA. For the dose sparing experiments with equivalent aluminum masses, each dosage included 50 µg of NPs alongside 25 µg OVA for DUT-5 and 50 µg of NPs alongside 25 µg OVA for alum. A summary of information regarding the dosage amounts and groups for these studies are summarized in Table 1. Equivalent prime and boost doses were delivered on day 0 and 14. 28 days after the initial dosage (14 days after the booster dosage), the mice were euthanized, blood and spleens were collected, and BAL was performed. From the blood, serum was collected following centrifugation at 1.5 K RCF for 10 min and was used to determine serum titers of IgG via indirect enzyme-linked immunosorbent assay (ELISA). Similarly, the supernatant from the BALF was collected following centrifugation at 0.5 K RCF for 5 min and was utilized to determine IgA titers via indirect ELISA.

The protocol for the indirect ELISAs were identical except for the secondary antibody, which, to detect IgG, was rat anti-mouse IgG-HRP (Southern Biotech), for IgA was goat-anti-mouse IgA-HRP (Southern Biotech), for IgG1 was rat anti-mouse IgG1-HRP (Southern Biotech), and for IgG2a was rat anti-mouse IgG2a-HRP (Southern Biotech). To prepare indirect ELISAs, 25 µg/mL OVA in 10 mM carbonate/bicarbonate buffer (pH 9.6) was added to a high binding 96-well plate (Corning) overnight at 4 °C. The plate was then washed three times using 1 × PBS with 0.05% by volume tween 20 (wash buffer). Plates were then soaked with 10% FACS buffer (10% FBS, 90% by volume PBS 1 ×) with 100 uL per well at 4 C for 1 h then washed again three times. Plates were then dried and samples at various serial dilutions (10 × dilution to 10^10^ × dilution) at 100 µL per well were added. For dilutions, 10% FACS buffer (10% FBS, 90% by volume PBS 1 ×) was used. Plates were then incubated at 37 °C for 2 h, washed 3 × with wash buffer, and 100 µL of anti-mouse antibodies with HRP conjugated was added per well at 1:4000 dilution. Plates were then incubated at 37 °C for 2 h and then washed 5 × with wash buffer. Then 100 µL of tetramethylbenzidine (TMB) solution (BD Biosciences) was added to each well and allowed to develop in the dark for 15 min. After, 100 µL of stop solution (2 N H_2_SO_4_) was added to each well and the absorbance of each well was read at 450 nm with background subtraction at 570 nm using BioTek Cytation5 Multimodal Plate Reader. Limit of detection for determination of titers was ascertained using absorbance values from PBS-dosed mouse samples. The average of these absorbance values for each of the respective antibodies was denoted as background and a cutoff value for titers was established as 2.5 × this baseline value [25]. The reported titer is the titer for which each of the vaccinated mouse samples reached 2.5 × the established baseline value.

### Histology

To assess acute responses, mice were euthanized 24 h after a single NP administration, tracheas were cannulated, and lungs filled with 50% OCT in PBS to fully inflate the airspace. The lungs were harvested and flash frozen in liquid nitrogen to preserve the lung structure. For histological analysis, the lungs were embedded in paraffin and cut at 7 μm sections. Sections were mounted to glass slides and stained using H&E stain. The sections were imaged using BioTek Cytation 5 Multimode Imager.

### In vivo murine bio distribution studies

To assess aluminum biodistribution, 4-week-old female C57BL/6 J mice were dosed with 100 µg of NPs alongside 25 µg OVA via orotracheal instillation with concentrations determined via TGA with groups and study details shown in Table 1. At 24 h post initial dosage and 28-day post initial dosage (14 days after booster dosage) time points, mice were euthanized, blood was collected by blood draw from vena cava, and organs were harvested and weighed. Organs were then minced and digested for 24 h in trace metal grade nitric acid (1 mL for heart, lungs, kidneys, spleen, and blood, 2 mL for liver, Fisher Scientific) at 75 °C. The resulting digested organs and blood were then filtered through a 0.22 um filter, diluted to 3% nitric acid in DI water from a MilliQ DI water system, and analyzed for their aluminum content via ICP-MS (inductively-coupled plasma mass spectrometry) using an Agilent 7500 ICP/MS.

### Statistics

GraphPad Prism 9 (GraphPad Software Inc) was used to perform statistical analysis. Figure captions denote the statistical tests used to carry out the analysis. All quantitative data are represented as mean ± SD (standard deviation) or SEM (standard error of the mean), as indicated in the figure caption as are the number of replicates. Tukey’s multiple comparisons test as part of one-way ANOVAs or Student’s T-test were used to generate p-values unless stated otherwise. Results shown are representative of at least two independent experiments, with particle or biological replicates reported in the figure captions.

## Results and discussion

### Negatively charged Al-based MOFs vary in particle size, but have aerodynamic sizes for effective alveolar deposition

**Fig. 1:**
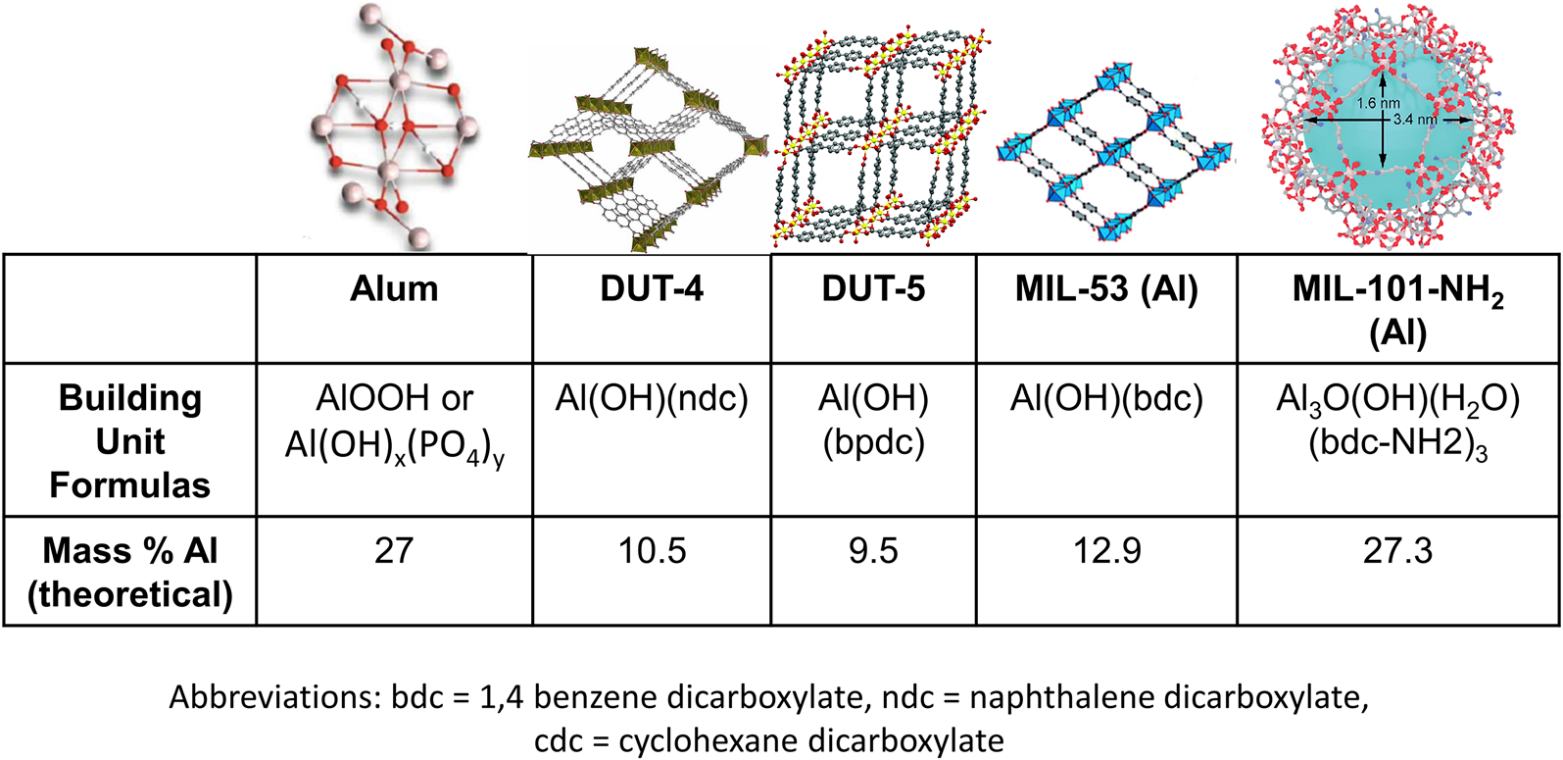
Comparison of Al-based NPs used in this work. Structures, chemical building formulas, and theoretical mass percentages of aluminum in the five Al-based NPs including the positive control, alum, as well as the four Al-based MOFs, DUT-4, DUT-5, MIL-53 (Al), and MIL-101-NH_2_ (Al). Alum image reproduced from Ref. [26] under open access license. DUT-4 image reproduced from Ref. [20]. MIL-53 (Al) image reproduced from Ref. [27], DUT-5 image reproduced from Ref. [28], MIL-101-NH2 (Al) image reproduced from Ref. [29] with permission from the Royal Society of Chemistry

The MOF NPs chosen for study were selected on the basis of the chemical structure of their metal clusters. In particular, all of the MOF NPs utilized have aluminum oxide/hydroxide-containing structures, similar to the chemical composition of alum [16]. Alum is often composed of aluminum oxyhydroxide (AlOOH) or aluminum hydroxyphosphate (Al(OH)_x_(PO_4_)_y_) [10, 11]. Figure 1 shows the structures and approximate theoretical Al content (by mass) of all five particle types utilized including alum, the positive control for an immunogenic, Al-containing adjuvant. As shown in the figure, the structures of the four MOFs synthesized, DUT-4, DUT-5, MIL-53 (Al), and MIL-101-NH_2_ (Al), all have motifs of AlO or AlOH in their metal clusters connected by various carboxylate ligands. Throughout this work, the grouping of the five NPs including alum will be referred to as “Al-based NPs,” while the grouping of the four MOFs excluding alum will be referred to as “Al-based MOFs.” Al-based MOFs were successfully fabricated per established protocols [20–22] and presented with expected crystalline structures and gas adsorption characteristics (Additional file 1: Figs. S1–S7).

**Fig. 2:**
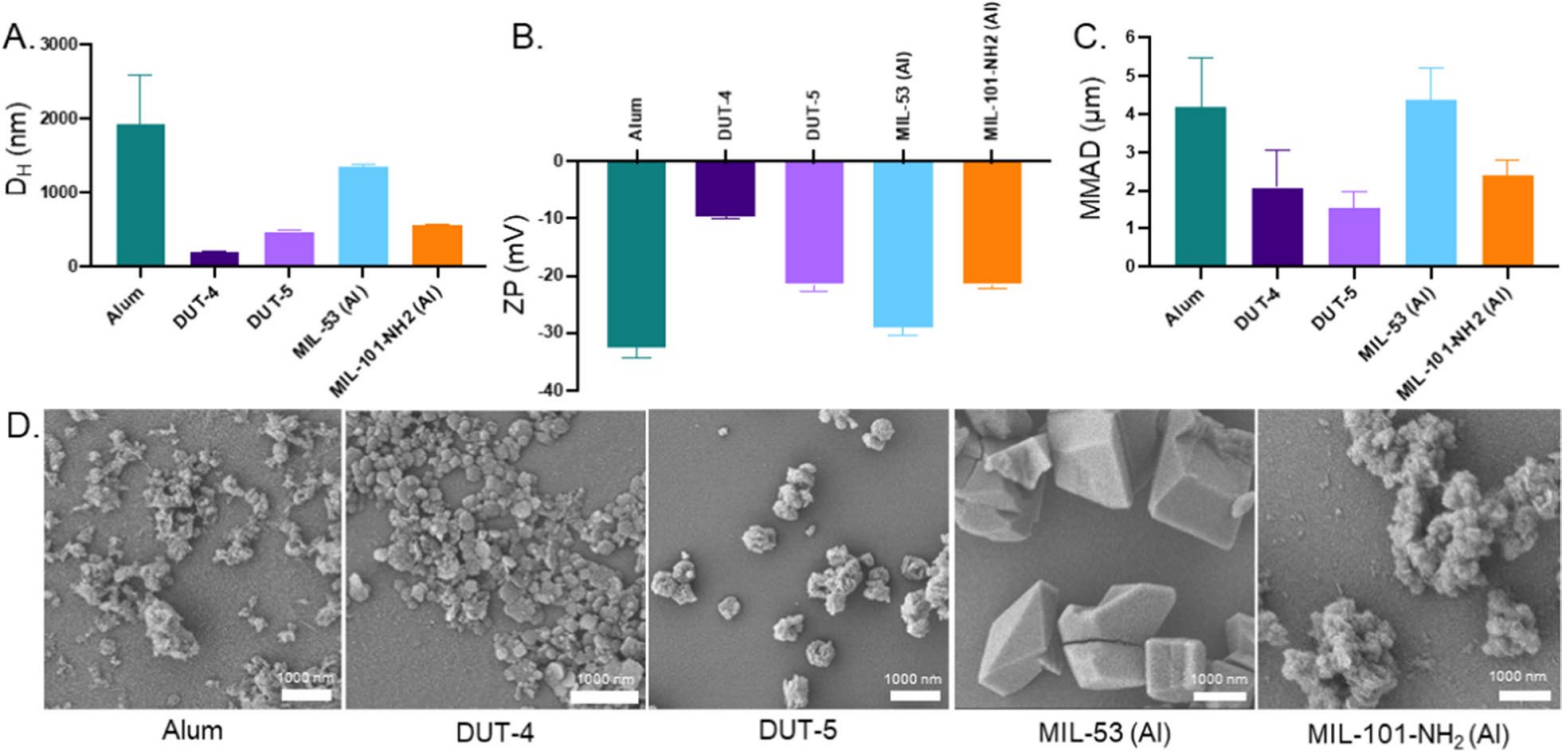
Size, shape, and charge characterization of Al-based NPs. **A** Size characterization of the Al NPs via dynamic light scattering (DLS) (*N* = 3). **B** Charge characterization of Al NPs via zeta potential (*N* = 3). **C** Mass median aerodynamic diameters (MMADs) of the five Al-based NPs evaluated from a PlastiApe Monodose dry powder inhaler on a Next Generation Impactor (NGI) at 60 L/min (*N=3*). **D** Scanning electron microscopy (SEM) images of Al NPs, showing their differing shapes and geometric sizes (all scale bars indicate 1000 nm). Data in **A**, **B**, and **C** represent mean with error bars for SD

While the MOFs have similar Al motifs in their clusters, the Al content of these NPs varies greatly, which may be a critical factor to examine in their adjuvanticity, alongside other NP properties such as size, charge, and shape that can affect uptake by target cells such as APCs and can also be connected to differences in their immunological effects [30–33]. These properties for as synthesized NPs were measured for all five NPs as synthesized, shown in Fig. 2 and summarized in Additional file 1: Table S1. Both the hydrodynamic (D_H_, Fig. 2a) and geometric sizes (D_G_, Additional file 1: Table S1) of the NPs vary greatly. Geometrically, the NPs vary in size from 200 nm for the DUT-4 NPs to over 1 µm for the MIL-53 (Al) NPs (Fig. 2d).

This difference in particle size (both D_H_ and D_G_) among the NP series may affect their uptake mechanism, as particles around 200 nm tend to be taken up via various types of endocytosis including phagocytosis, while 1 µm particles will be predominantly take up by APCs via phagocytosis, not via mechanisms such as receptor-mediated endocytosis [30]. It has also been shown that particle size has significant effects on the adjuvanticity of various particles. For example, for poly(methyl methacrylate) (PMMA) and polystyrene (PS) particles, it was shown that smaller NPs (~ 60 nm) had greater adjuvanticity than larger NPs (~ 300 nm) [34], while other studies on PLGA NPs found that the optimal size for NPs for adjuvanticity was ~ 900 nm (relative to particles on the size of 500 nm to almost 5 μm) [35]. A potentially more relevant comparison using alum determined that alum processed to be more nanoparticulate in size (~ 70 nm) had greater adjuvanticity and more Th1 character than the typical ~ micron-sized alum [36]. The particles utilized in this study fall in this range, though larger than 70 nm, which may provide insights on differences in adjuvanticity between particle types.

While the sizes of the NPs do vary greatly, the charge of the NPs (as measured via zeta potential, ZP, and shown in Fig. 2b) are all negatively charged on the order of − 10 mV. The alum NPs have the greatest charge magnitude of − 32.6 ± 1.9 mV while the DUT-4 have the lowest charge magnitude of − 9.7 ± 0.2 mV. The other three NPs, DUT-5, MIL-53 (Al), and MIL-101-NH_2_ (Al), have charges between − 20 and − 30 mV on average, which means that they, alongside the alum, will have effective charge stabilization in solution and are less likely to aggregate, whereas the DUT-4 NPs, which have a statistically significantly lower magnitude of charge than the other NP types, are more likely to be prone to aggregation because of less interparticle repulsion [37]. The ZP of these NPs being negative is also expected given the surface display of negatively charged carboxylate ligands in the case of the MOF NPs and the hydroxyl groups on the surface of the alum. Other studies have demonstrated that there may be benefits to positive overall charge in terms of greater relative uptake in lung APCs because of the electrostatic attraction exhibited between positively charged NPs and the negative charge of cell membranes [31, 38]; however, cationic NPs also tended to exhibit greater cytotoxicity relative to their negatively charged counterparts [39].While the charge of NPs can influence antigen association, APC uptake, and tissue distribution, since the NPs studied all have slightly negative charges, this factor of charge will likely not have a significant influence on the relative adjuvanticity of the respective Al-based MOF NPs [36].

To better understand the expected deposition in the lung of the Al-based MOFs, especially relative to alum, we formulated the particles into dry powders via lyophilization and determined their mass median aerodynamic diameters (MMADs) via deposition following aerosolization in a Copley Next Generation Impactor (NGI). The results of these aerosolization studies, shown in Fig. 2c, demonstrate that DUT-4, DUT-5, and MIL-101-NH_2_ (Al) all have MMADs falling in the range of 1.5–2.5 µm, making them the most ideal candidates for pulmonary vaccination. Efficient peripheral airway deposition occurs for particles with MMAD between 1 and 3 µm in size, with expected deposition efficiencies greater than 80% [40]. Outside of this range, deposition rates rapidly fall, particularly for particles with MMAD below 0.75 µm in size or above 5 µm in size. The alum and MIL-53 (Al) particles had MMADs around 4 µm, which would correspond with expected alveolar deposition rates of ~ 65% based on this analysis [40]. It is important to note, however, that the sizes and expected deposition rates reported here apply only to the aggregated particles as dispersed by the Monodose inhaler used and assumes constant inhalation rate. A wide variety of inhaler types and aggregation states could be utilized for delivery of these particles, and thus, future analyses should consider varying these factors as well as different patient lung geometries and disease states, which will affect deposition as well [41, 42].

### Al-based MOFs demonstrate promising in vitro biocompatibility, cellular uptake, and co-stimulatory response of APCs

**Fig. 3:**
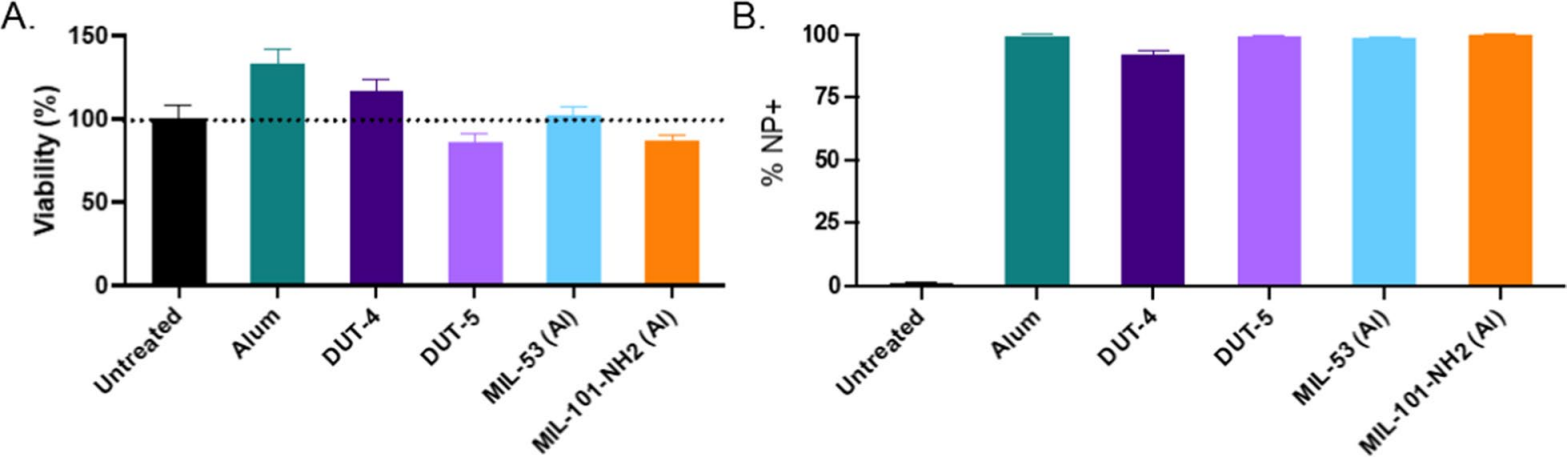
Viability and cellular uptake of Al-based NPs by RAW264.7 cells 24 h after dosage. For uptake, all Al-based NPs were loaded with FITC, and uptake determined via flow cytometry (*N* = 3). All samples were treated with trypan blue prior to flow cytometric analysis or imaging to quench external FITC signal. **A** Cell viability following dosage with Al-based NPs at 100 µg/mL. **B** Percent of cells that were positive for particle uptake as determined via flow cytometry. Data represent means with error bars for SD

The candidate Al-based MOFs, alongside alum, were evaluated for their acute in vitro cytotoxicity and cell uptake as a potential explanation for differences observed in later in vitro and in vivo evaluations. NP in vitro biocompatibility was tested via dosage to a cell line of RAW264.7 macrophages as a representative APC and one of the key cellular targets for in vivo vaccination, as macrophages aid in beginning the education of other immune cells and generation of a potent immune response if activated [14, 43]. As shown in Fig. 3a, all five Al-based NPs indicate high cell viability (normalized to that of untreated cells), which was confirmed to be non-statistically different than the untreated via Tukey’s multiple comparisons test following analysis from a one-way ANOVA. The plot shows viability for cells after 24 h when dosed with NPs at a final concentration of 100 µg/mL for each of the Al-based NPs (amounts, times, dosage, and N also summarized in Table 1), which showed a similar lack of cytotoxicity following dosage at 10 µg/mL as well (Additional file 1: Fig. S8). Both alum and DUT-4 show slight increases in observed viability relative to the untreated RAW cells, which may indicate a slight increase in metabolic activity; however, this increase was not statistically significant.

Shown in Fig. 3b, uptake of RAW cells dosed with 100 µg/mL of each of the Al-based NPs (pre-loaded with FITC for tracking uptake) showed near 100% uptake of the NPs 24 h after dosage. This uptake was determined via flow cytometry (representative gating shown in Additional file 1: Fig. S9) following dosage of FITC-loaded NPs and trypan blue staining to quench signal from external FITC on the surface of the cells (representative images shown in Additional file 1: Fig. S10), which would also be expected to be minimal based on prior results with similarly structured MOFs [18]. Thus, despite noticeable differences in overall size, which may affect the route of uptake and subsequently the rate of cargo release, NPs were internalized at statistically equivalent extents in vitro, indicating that any phenotypical differences observed in vitro are not a result from differential uptake of the different NPs.

**Fig. 4:**
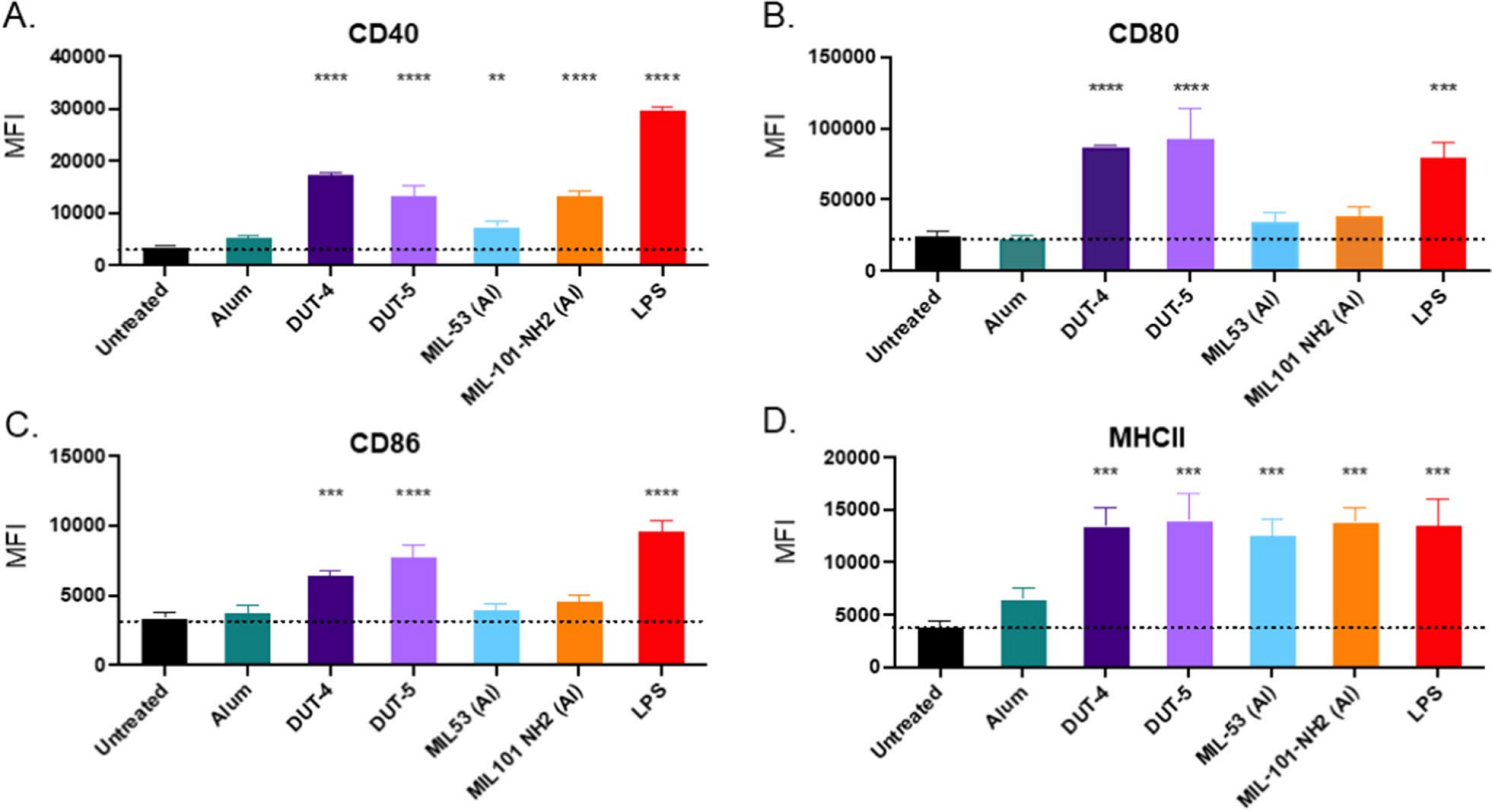
Upregulation of various surface markers for RAW264.7 cells by Al-based NPs and LPS as potential indicators of future stimulation of APCs in vivo. The figure shows median fluorescence intensity (MFI) of **A** CD40, **B** CD80, **C** CD86, and **D** MHC II as determined by flow cytometry for RAW cells dosed with various NPs at 100 µg/mL or LPS at 10 ng/mL (*N* = 3). Statistics shown are comparisons of each group relative to the untreated control (ns *p* > 0.05, *p < 0.05, **p < 0.01, ***p < 0.001, ****p < 0.0001) as determined via Tukey’s multiple comparisons test as part of a one-way ANOVA. Data represent mean with error bars for SD

Given the lack of cytotoxicity and high uptake of the Al-based NPs, we then determined the extent to which the NPs could cause activation of macrophages in vitro, which was measured via upregulation of co-stimulatory cell surface markers, namely CD40 (cluster of differentiation 40), CD80, CD86, and MHC II. As with the studies to investigate cytotoxicity, RAW264.7 macrophages were dosed with 100 µg/mL of each of the five respective Al-based NPs (amounts, times, dosage, and replicates also summarized in Table 1) alongside a treatment of LPS of 10 ng/mL as a positive control for stimulation of the immune cells. As shown in Fig. 4, both DUT-4 and DUT-5 had statistically significant upregulation of all four co-stimulatory markers relative to the untreated control and also relative to alum. Macrophages treated with DUT-4 had high expression of all four co-stimulatory markers, exceeding those of the untreated macrophages by at least 90% (CD86) and up to almost 400% greater expression for CD40. In fact, upregulation of these markers was often comparable to the expression for the positive control, LPS, particularly for CD80 and MHC II. This was the case for macrophages treated with DUT-5 as well, whose expression of these co-stimulatory markers was very similar to those treated with DUT-4 and similar to the expression of cells treated with LPS. On the other hand, MIL-53 (Al) and MIL-101-NH_2_ (Al) tended to have lower relative extents of surface marker upregulation, only statistically exceeding that of the untreated macrophages in the cases of CD40 and MHC II, which may indicate lesser ability to activate APCs. In those cases, they exceeded the expression of CD40 by 112% and 282%, respectively, relative to the untreated cells and MHC II by over 220% as well. Unlike the macrophages treated with Al-based MOFs, macrophages treated with alum did not statistically differ from untreated macrophages. This is likely because of the hypothesized mechanisms of actions for alum’s adjuvanticity: the formation of depots in vivo for gradual release of antigen, destabilization of antigens for easy processing, and effective co-localization of antigen with adjuvant, all of which would have weaker or no effects in vitro and is consistent with prior reports [11, 13, 44].

Observed differences in macrophage activation could be attributed to many factors, including differences in particle sizes. The differences in particle size could lead to different routes of uptake, as the smaller NPs, namely DUT-4 and DUT-5, are more likely to be internalized via endocytosis than the particles that are micron sized or larger [30, 31]. The differences in activation can also potentially arise from variations in the chemical compositions of the respective NPs. Structurally, the metal clusters of the MOFs are very similar, all having Al(OH)-based clusters with the slight exception of MIL-101-NH_2_ (Al), whose metal cluster also has coordinated oxides in addition to hydroxides, which may influence its adjuvanticity [20–22, 45]. The other key differences structurally are the length of the carboxylate-based ligands for DUT-4 and DUT-5 relative to MIL-53 (Al) and MIL-101-NH_2_ (Al), as DUT-4 and DUT-5 have longer linkers in the form of naphthalene and biphenyl dicarboxylates instead of the shorter benzene dicarboxylate ligand found in both MIL MOFs. This may allow for more rapid diffusion of compounds capable of breaking down the MOFs due to reduced steric hindrance, causing more rapid breakdown of the DUT MOFs, increasing their activation of RAW macrophages on the 24 h timescale [46].

### In vivo pulmonary murine vaccination demonstrates robust humoral response from all Al-based MOFs locally and systemically

Given the low cytotoxicity and high uptake of all of the Al-based NPs in vitro, we next tested their adjuvant properties in vivo through the generation of antigen-specific antibody titers and stimulation of pulmonary APCs. Female C57Bl/6 mice were immunized with a prime and boost dosage of 100 µg NPs alongside 25 µg of ovalbumin (OVA), a well-studied model antigen for mouse models, at 0-day and 14-day time points via an orotracheal administration. In this immunization, we fixed the total mass of particles delivered, leading to equivalent NP mass delivered directly to the lung (amounts, times, dosage, and N also summarized in Table 1). For these immunizations, no additional adjuvant was added to the formulations; endotoxin-free OVA was used so that only the respective particles could act as adjuvants.

**Fig. 5:**
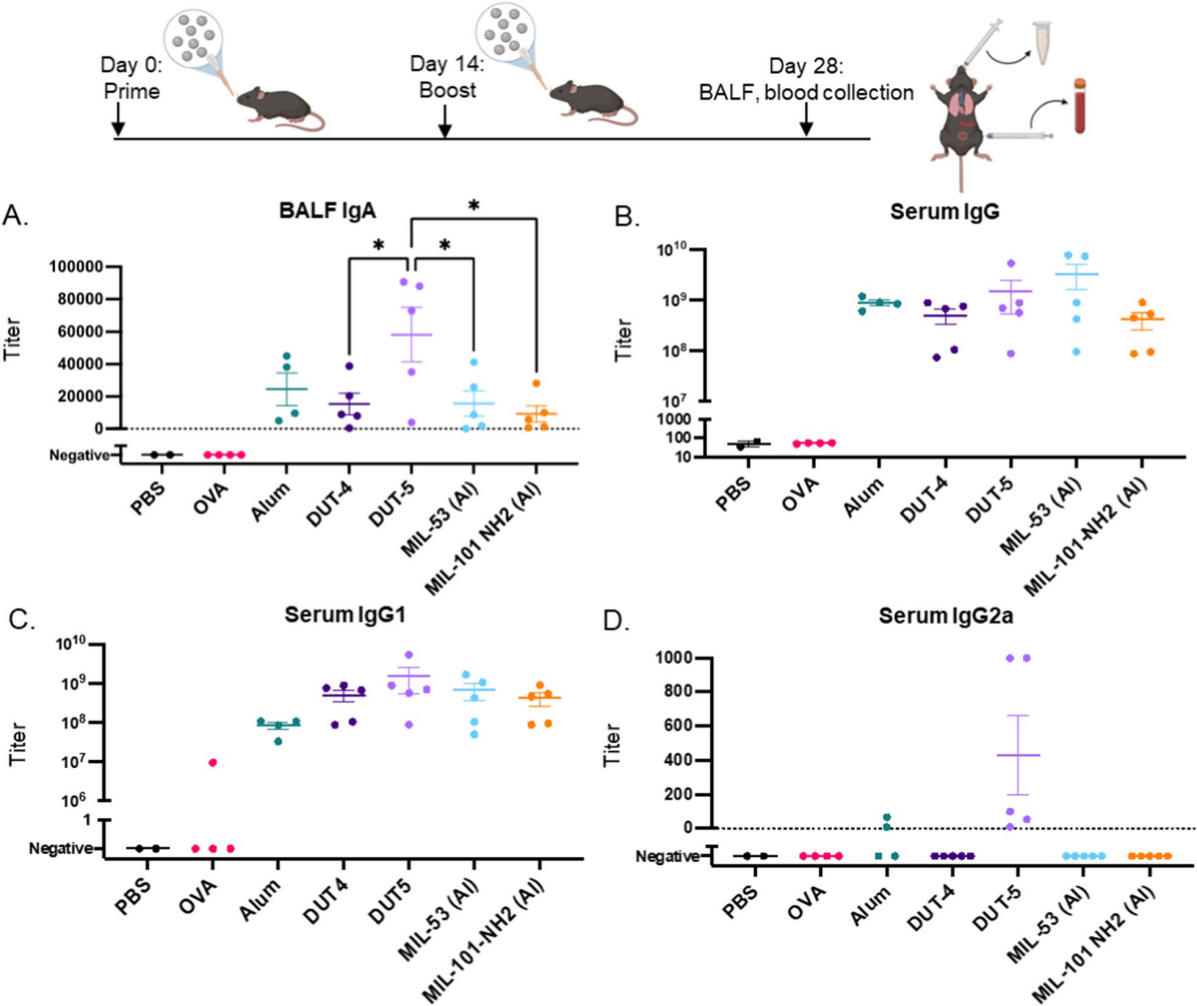
Pulmonary murine vaccination study with AL-based NPs. Antibody titers for mice vaccinated with various Al-based NPs and OVA (prime and boost) from day 28 after initial dosage. **A** BALF IgA titers. **B** Serum IgG titers. **C** Serum IgG1 titers. **D** Serum IgG2a titers. Statistics shown are comparisons of each group relative to the other groups (*p < 0.05) and where not shown indicates no statistically significant difference via Tukey’s multiple comparison tests as part of a one-way ANOVA. Analyses were performed excluding “negative” results as they lack variance. Data bars represent the mean with SEM.

Following pulmonary vaccination, we measured IgA localized to the lung mucosa to detect development of local immunity and IgG in the serum to detect systemic immunity, both of which are critical for protection against future infection [4, 47]. Accordingly, we determined the antibody titers against OVA for all of the Al-based MOF vaccinations (MOFs + OVA) in addition to those of PBS-dosed and OVA-dosed mice as well as Alum + OVA. The resulting antibody titers (Fig. 5) demonstrate robust generation of both local (IgA) and systemic (IgG) antibodies. Overall, Al-based MOFs dosed at equivalent particle masses yielded both OVA-specific IgG and IgA titers on par or greater than those of alum, as discussed in the following paragraphs.

For IgA, all dosage groups exhibited average titers on the order of 10^4^, while mice dosed with PBS only or OVA only did not have detectable antibody titers against OVA, as shown in Fig. 5a. Because of the variability of the titers, none of the IgA titers were statistically significantly different than those from the treatment with alum as evaluated via Tukey’s multiple comparisons as part of a one-way ANOVA; however, DUT-5 did have statistically greater IgA titers than the other three Al-based MOFs, indicating that it has the greatest ability to generate a local antibody response in vivo.

The development of IgA tiers is a critical aspect of mucosal protection, which must be initiated through class switching of B cells, has been demonstrated to be essential for the effective protection of mucosal surfaces against airborne pathogens [47, 48]. The presence of IgA antibodies effectively prevents pathogen entry into cells, opsonizes the pathogens for clearance by phagocytic cells, and also allows for more ready removal via the mucociliary escalator [47, 49, 50]. Secretion of IgA at the mucosa has also been shown to neutralize pathogens more effectively than IgG antibodies despite their smaller numbers and also correlates with greater vaccine efficacy (particularly for influenza) [51–53]. The IgA antibody titers from vaccination with Al-based MOFs (especially DUT-5) were, in some cases, more than an order of magnitude higher than IgA titers from other murine investigations of mucosal vaccination with OVA, as well as dosage of OVA alone in this study (IgA and IgG titers indistinguishable from those of PBS-dosed mice), indicating the potent mucosal protection afforded via pulmonary vaccination with Al-based MOFs [54, 55].

This resultant higher IgA antibody titers for DUT-5 could be a function a greater ability to activate APCs (in line with in vitro results for macrophages in Fig. 4), which cascades to greater B cell activation and antibody production. This may be an explanation for its higher antibody titers compared to alum, MIL-53 (Al), and MIL-101-NH_2_ (Al); however, given that DUT-4, which had similar activation of macrophages in vitro (Fig. 4), did not achieve statistically equivalent levels of IgA production (Fig. 5a), APC activation alone likely cannot explain the difference in antibody titers. Differential uptake in vivo (which can differ from uptake in vitro) coupled with more effective in vivo APC activation could be responsible for the significant increase in IgA antibodies for DUT-5. Because of DUT-4’s smaller size, it is more likely to undergo uptake via endocytic pathways instead of phagocytic ones, and previous studies using NPs of similar charge and differing in size demonstrated that murine macrophages favored uptake of ~ 500 nm NPs (similar in size to DUT-5) relative to the uptake of ~ 150 nm NPs (similar in size to DUT-4 NPs) [31, 39]. DUT-4 may also lead to lower stimulation of each APC because of its lower mass per particle relative to DUT-5, which is likely to be a greater factor in vivo, as in vitro dosage led to ~ 100% uptake. DUT-5’s more effective IgA antibody generation than the other MOFs could also indicate that, for a fixed mass dosage, there is an optimal particle size to balance stimulation of individual APCs (potentially a critical mass of Al delivered) alongside stimulation of a large enough number of APCs.

Interestingly, all Al-based NPs were roughly equivalently effective at generating total serum IgG (Fig. 5b), with no titer being statistically significantly higher than any other treatment as determined via Tukey’s multiple comparisons test as part of a one-way ANOVA. The MOFs all had antibody titers comparable to those of mice vaccinated with alum, indicating their equivalent adjuvanticity for this dosage. Despite greater IgA titers, DUT-5 did not generate higher levels of IgG antibodies than the other treatments, which may indicate that the dosage of Al NPs has exceeded the dose required to generate an antibody response of this magnitude for all of the respective Al NPs.

Further investigation into the IgG subtypes was performed to characterize the nature of the immune response generated by the different Al-based NPs. Serum antibody titers of IgG1, the IgG subclass more commonly associated with Th2 responses, and IgG2a, the IgG subclass more commonly associated with Th1 responses, were determined for each of the vaccinations with the Al-based NPs (Fig. 5c, d) [56]. Previous studies with alum have demonstrated that alum typically generates IgG1 antibodies, which is similar for all of the Al-based MOFs, as well, indicating that they will drive predominantly humoral, antibody-driven responses to protect from invasion from pathogens (Fig. 5c) [57]. Similar to IgG titers, the IgG1 titers of the Al-based MOFs were not statistically significantly different from those of alum, indicating that all NPs drive a strong humoral response.

In line with robust IgA antibody generation, DUT-5 was the only vaccination treatment that led to observable IgG2a antibody production for all mice dosed. DUT-5 had IgG2a antibody titers averaging over 400 with detectable IgG2a antibodies from all mice, while all other Al-based NP vaccines did not have detectable IgG2a antibodies apart from two mice vaccinated with alum, both of which had antibody titers well below 100. These differences were not statistically significant; however, because of lack of variability among the vaccinations with no detectable titers (shown as “Negative” in Fig. 5d). This could indicate that DUT-5 can uniquely drive a more effectively generate mucosal response by engendering both humoral and cell-mediated immunity; however, these overall titers are significantly lower than strong Th1 adjuvants. Further studies with greater replicates could also be performed to further confirm these results.

### Equivalent Al vaccination dosing demonstrates superior adjuvanticity of Al-based MOFs

**Fig. 6:**
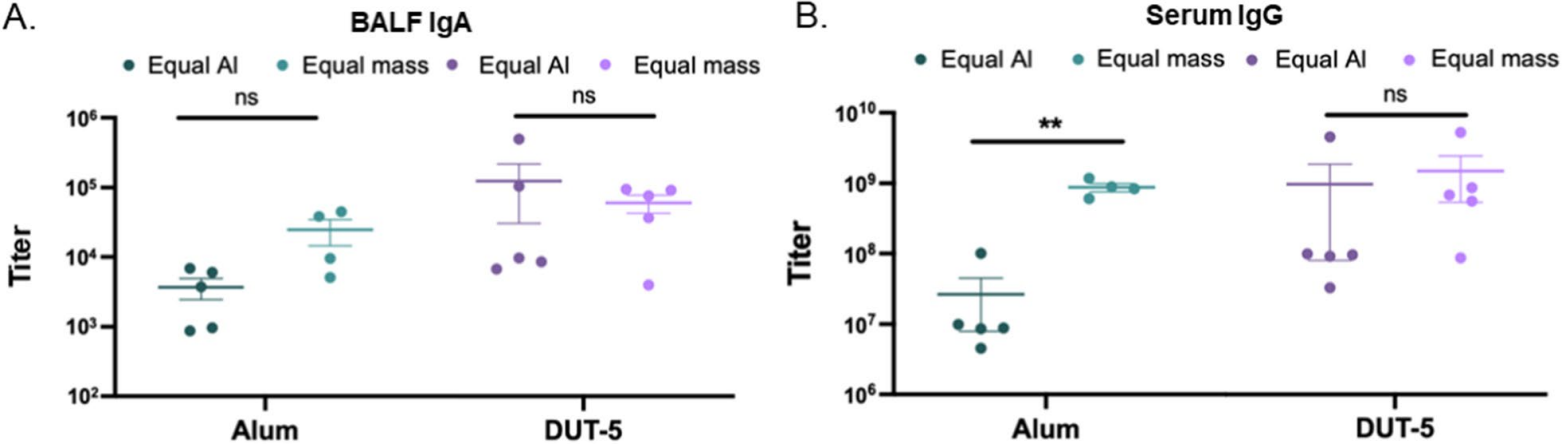
Pulmonary murine vaccination at equivalent Al and reduced mass. Antibody titers for mice vaccinated with alum or DUT-5 and OVA (prime and boost) with reduced dosage of particles from day 28 after initial dosage (equal Al) versus antibody titers from original dosage (equal mass) reproduced from Fig. 5. **A** Comparison between BALF IgA titers from equal mass (original dosage, *N* = 4 for alum, *N* = 5 for DUT-5) and equal Al (reduced dosage, *N* = 5 for alum and DUT-5) experiments **B** Comparison between serum IgG titers from equal mass (original dosage) and equal Al (reduced dosage) experiments. Statistics shown are from paired *t* tests between the equal mass (original from Fig. 5) and equal Al (reduced dosage) experiments for each of the respective groups with **p < 0.01

In the prior studies, NPs were all dosed at equivalent NP mass, leading to potentially different amounts of Al. To test the hypothesis that MOFs could provide more effective immunization at lower Al content, we next explored dosing at equivalent Al concentrations to directly compare the adjuvanticity of DUT-5 with alum. We also reduced the dosage of DUT-5 (with alum dosage adjusted accordingly to equivalent Al concentration) to determine whether the prior dosage of 100 µg/mouse exceeded the required dosage to achieve the previously observed IgG and IgA titers (amounts, times, dosage, and N also summarized in Table 1). The results of this experiment plotted alongside those from our prior study are shown in Fig. 6 with direct comparisons between alum and DUT-5 in Additional file 1: Fig. S11. Even though mice in this study were vaccinated with 50% of the dosage of DUT-5 relative to those in the original vaccination at 100 µg/mouse (Fig. 5), the mice from the reduced dosage (Equal Al) had statistically equivalent antibody titers for IgA (Fig. 6a) and IgG (Fig. 6b) as determined via a paired *t*-test. This indicates that the previous hypothesis that the original dosage may have exceeded the required dosage to maximize antibody titers was correct for IgA and IgG; however, the 50% dosage of DUT-5 did not have detectable IgG2a titers, indicating that the higher dosage (100 µg/mouse) may be necessary for effectively stimulating a cellular response, while the generation of the humoral response, which was approximately equivalent, does not require the greater dosage. This was not the case for alum-vaccinated mice, whose IgG (Fig. 6b) titers were statistically significantly reduced relative to the original, higher dosage and thus are dose-dependent for the range studied. While not statistically significant (*p* = 0.13), there was also a reduction in observed IgA titers (Fig. 6a).

Furthermore, these results excitingly indicate that DUT-5-vaccinated mice vastly outperform alum-vaccinated mice in terms of generation of antibody titers at equivalent aluminum content. Average IgA (Fig. 6a, Additional file 1: Fig. S11A) and IgG (Fig. 6b, Additional file 1: Fig. S11B) titers for mice vaccinated with DUT-5 exceed those for equivalently-dosed alum-vaccinated mice by more than an order of magnitude (~ 30 × greater titers for both classes of antibodies). For IgG, mice vaccinated with alum have titers of 2.65 × 10^8^ ± 1.85 × 10^8^ while mice vaccinated with DUT-5 have titers of 9.87 × 10^8^ ± 9.06 × 10^8^. For IgA, mice vaccinated with alum have titers of 3.69 × 10^3^ ± 1.24 × 10^3^ while mice vaccinated with DUT-5 have titers of 1.18 × 10^5^ ± 8.92 × 10^4^.

These results demonstrate the effectiveness of DUT-5 at equivalent aluminum content, indicating its great potential as an adjuvant for pulmonary vaccination. Given the equivalent Al content, the greater titers of DUT-5 relative to alum may indicate that particle number is critical for effective immune stimulation. Alum will have fewer particles when dosed at either equivalent mass of NP or equivalent Al content; alum NPs have a larger average diameter than DUT-5 NPs and also have a greater relative percentage of aluminum (approximate particle numbers are shown in Additional file 1: Table S2). In tandem with the greater IgA titers of DUT-5 from the vaccination at 100 µg/mouse (Fig. 5a) relative to the other Al-based MOFs, these results suggest that both particle number and aluminum content per particle may be critical parameters for maximizing antibody titers using aluminum-based adjuvants. DUT-5, at an intermediate particle size (between 200 nm of DUT-4 and 1000 + nm of the MIL MOFs) and lower aluminum content relative to MIL-53 (Al) and MIL-101-NH_2_ (Al) will have a greater number of particles (Table 2) available for uptake and stimulation of APCs than the MIL MOFs. This brings us to the question of DUT-4’s lower IgA titers; if particle number were the only critical parameter for stimulation of the immune system (leading to greater titers), then DUT-4 would be expected to perform better than DUT-5. Since the IgA titers are statistically lower for mice vaccinated with DUT-4 than for those vaccinated with DUT-5, there is likely a critical amount of Al necessary for individual APC activation, which cascades to greater IgA titers. Another hypothesis for this difference could be differential in vivo uptake, as discussed with regard to results in Fig. 5.

**Table 2:** Mass and approximate number of particles for in vivo studies with Al-based NPs

	Alum	DUT-4	DUT-5	MIL-53 (Al)	MIL-101 NH_2_ (Al)
In vivo NP mass dosage (equal mass studies)	100 µg/mouse	100 µg/mouse	100 µg/mouse	100 µg/mouse	100 µg/mouse
In vivo NP approx. number dosage (equal mass studies)	2.43 × 10^7^ particles/mouse	1.36 × 10^10^ particles/mouse	1.68 × 10^9^ particles/mouse	1.71 × 10^7^ particles/mouse	1.29 × 10^9^ particles/mouse
In vivo NP mass dosage (equal Al studies)	25 µg/mouse	N/A	50 µg/mouse	N/A	N/A
In vivo NP approx. number dosage (equal Al studies)	6.09 × 10^6^ particles/mouse	N/A	8.42 × 10^9^ particles/mouse	N/A	N/A

All in vivo studies (listed in Table 1) were “equal mass studies” with the exception of the “equal Al studies” described in this sub-section. Calculations for approximation of particle numbers can be found in the SI

### Al-based MOFs activate alveolar macrophages in vivo and demonstrate good safety via neutrophil recruitment and histological analysis

**Fig. 7:**
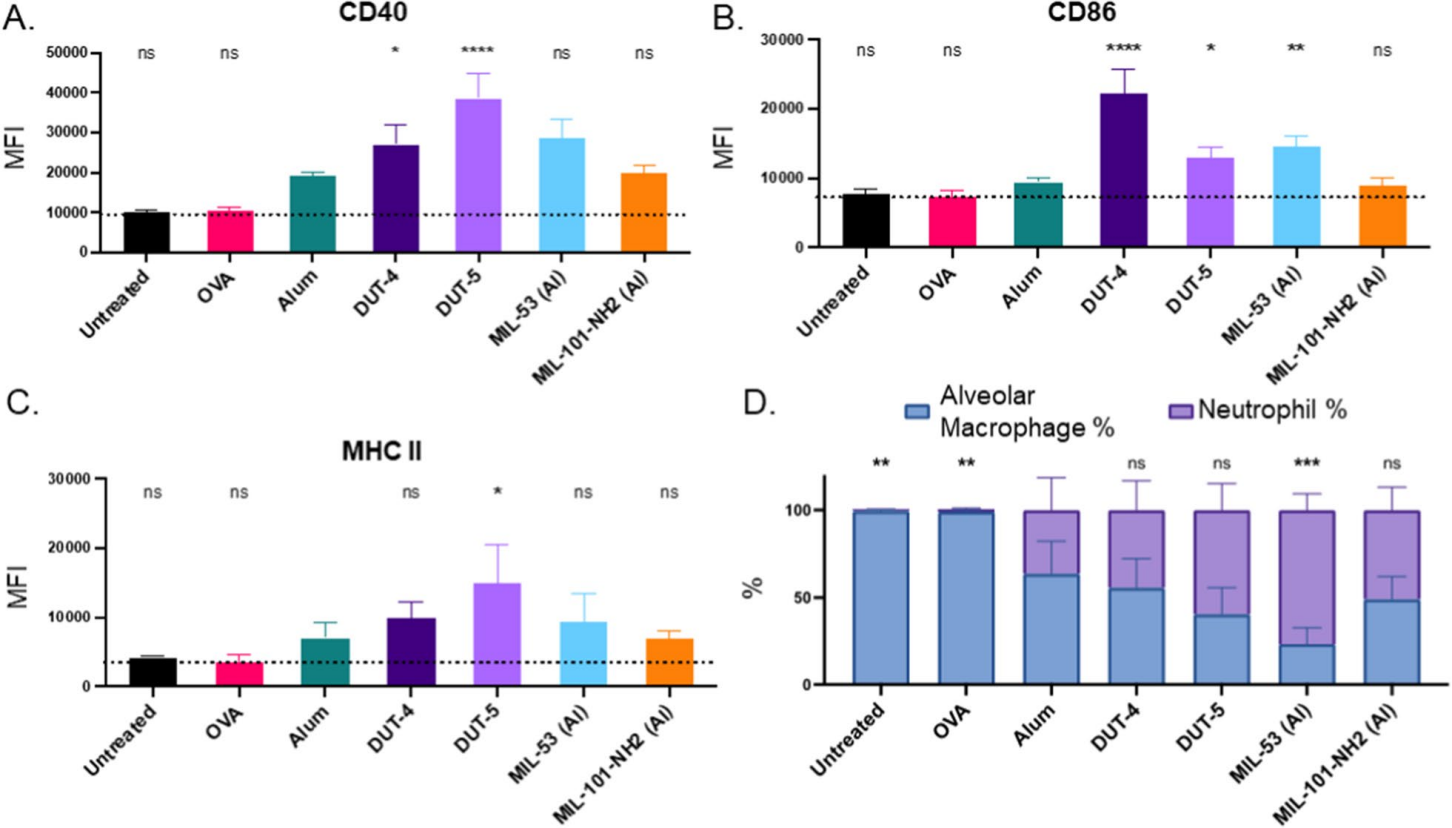
In vivo activation of alveolar macrophages and recruitment of innate immune cells 24 h after prime dosage. MFI of three co-stimulatory markers on the surfaces of alveolar macrophages normalized relative to the MFI of untreated mice **A** CD40, **B** CD86, and **C** MHC II. Statistical comparisons shown are comparisons to alum. **D** Percentages of alveolar macrophages and neutrophils recruited to the lungs and determined via flow cytometry (AMs were identified as CD45+, SiglecF+, and Ly6G−, while neutrophils were CD45+, Ly6G+, and SiglecF−). Statistics shown are comparisons of each group relative to the positive control (*p < 0.05, **p < 0.01, ***p < 0.001, ****p < 0.0001) and where shown as “ns” indicates no statistically significant difference relative to positive control via Tukey’s multiple comparison tests as part of a one-way ANOVA. (*N* = 2 for PBS, *N* = 4 for alum and OVA, *N* = 5 for all Al-based MOFs). Error bars indicate SEM

Given the promising vaccination results, we sought to determine the APC activation ability and tolerability of Al-MOF adjuvants in the lung. The acute, in vivo activation of APCs in the pulmonary space was determined 24 h after the prime dosage of the respective NPs to the mice following dosage of 100 µg of particles/mouse (amounts, times, dosage, and N also summarized in Table 1). Because the desired application for these adjuvants is in pulmonary vaccination, alveolar macrophages were identified via flow cytometry, and their activation was measured via upregulation of co-stimulatory surface markers following orotracheal instillation of the respective NPs. As shown in Fig. 7a–c, all of the Al-based NPs upregulated co-stimulatory markers relative to PBS-dosed mice. Despite its clinical relevance and use as a positive control for these studies, alum generally upregulated these markers less than the Al-based MOFs. In particular, DUT-4 and DUT-5 had statistically significant upregulation of both CD40 and CD80 relative to alum-dosed mice (as well as MHC II for DUT-5). On the other hand, MIL-53 (Al) and MIL-101-NH_2_ (Al) did not have statistically significant upregulation of any markers except for CD86 for MIL-53 (Al).

These results align well with the results of in vitro marker upregulation (Fig. 4), indicating that for these MOFs, in vitro activation may be an effective predictor of in vivo activation for macrophages. DUT-4 and DUT-5’s significant upregulation of these co-stimulatory markers in vivo indicates that these MOFs are more effective adjuvants in terms of APC activation, a critical first indicator of immunogenicity of the NPs. Activated APCs will educate T cells, beginning the cascade of effects leading to immune memory and antibody protection. These results may also indicate that in vivo APC activation can aid in prediction of local antibody generation, as DUT-5 had both the greatest generation of IgA antibodies and also the greatest upregulation of co-stimulatory markers for alveolar macrophages. As discussed in previously, other factors such as particle size may also be critical factors in the activation of APCs and generation of antibodies, as DUT-4 did not generate as robust of an IgA or IgG2a antibody response relative to DUT-5.

This set of results also gives more credence to the hypothesis that the amount of Al per NP as well as the number of NPs (Table 2) may be critical to both APC activation and to antibody generation, as DUT-5 had greater activation of APCs in vivo for CD40 and MHC II alongside greater IgA antibody titers relative to any of the other Al-based NP vaccinations. The greater activation of these APCs was achieved by DUT-4 dosing; however, it does not correlate with greater generation of antibody titers relative to alum, MIL-53 (Al), or MIL-101-NH_2_ (Al). This may indicate that other factors such as the depot effect may be less effective for a smaller NPs such as DUT-4 and potentially that it may have less effective adsorption of the antigen than the other Al-based NPs, leading to similar titers to alum, MIL-53 (Al), and MIL-101-NH_2_ (Al), despite increases in APC activation in vivo [5, 9, 58].

It is also worth noting, as shown in Fig. 7d, that all of the Al-MOFs (dosed at 100 µg of particles/mouse) recruit neutrophils to the lungs (as revealed via flow cytometry—alveolar macrophages were CD45+/SiglecF+/Ly6G−, while neutrophils were CD45+/Ly6G+/SiglecF−), while PBS dosed mice and OVA-dosed mice do not. Some neutrophil recruitment is advantageous, as neutrophils secrete cytokines and chemokines that attract more immune cells to the site of vaccination and can even act as APCs [44, 59]. This attraction can be critical for more effective education of T cells and B cells because more APCs with the desired antigen can drain to lymph nodes for adaptive immune cell education and antigen presentation. Though the recruitment aspect of neutrophils can be advantageous, they also have indiscriminate effects when recruited and can cause too much inflammation at the location to which they are recruited and cause tissue damage. All of the Al-based MOFs had neutrophil recruitment comparable to that of alum with the exception of MIL-53 (Al), which had statistically significantly higher recruitment to the lungs at this 24 h time point. Previous work has demonstrated that following alum vaccination, there is ~ 40–60-fold neutrophil recruitment to the site of dosage, which agrees well with the results found here and also aligns with the results for the Al-based MOFs with the exception of MIL-53 (Al), which has greater neutrophil recruitment [44]. The same work [44] demonstrated that, while neutrophils were partially responsible for relocation of the antigen to lymph nodes for T cell and B cell education, they were not necessary for the adjuvanticity of alum, but did enhance its immunogenicity. Similar studies regarding antigen trafficking could be beneficial for these Al-based MOFs as undue inflammation in the lungs, which can occur from great neutrophil recruitment, is a significant safety concern for pulmonary vaccination [17]. These results do suggest caution for further implementation and require follow-on studies of tolerability and overall pulmonary inflammation at varied Al-MOF dosages.

**Fig. 8:**
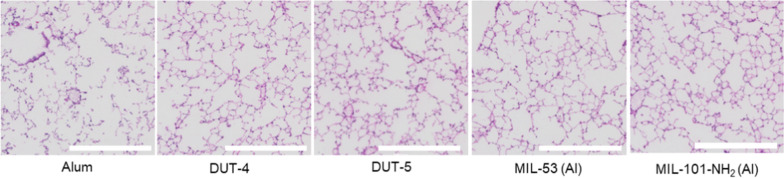
Histological analysis of murine lungs 24 h after prime dose. Representative lung sections from mice dosed with alum and the four respective Al-based MOFs, DUT-4, DUT-5, MIL-53 (Al), and MIL-101-NH_2_ (Al). The images were taken of the H&E-stained lung sections at a 4 × magnification and all have equivalent scale bars representing 500 µm. Image brightness was adjusted equivalently to all images in post processing

To better asses the safety of the Al-MOFs, histological analysis was performed with dosage of 100 µg of particles/mouse. The hematoxylin and eosin (H&E)-stained lung samples (4 × images shown in Fig. 8, 20 × images and additional controls can be found in Additional file 1: Fig. S12) were acquired 24 h after dosage with each of the respective Al-based NPs or PBS or OVA-only dosed mice. The images do not indicate any undue inflammation caused by the dosage of these NPs, as may have been expected given the high fraction of neutrophils observed (Fig. 7d). While there is some evidence of mild cellular infiltration, there is no evidence of alveolar wall thickening; thus, Al-based NP groups are largely indistinguishable from PBS-dosed mice, in terms of cellularity and low inflammation, resembling sections from biocompatible NPs [60, 61]. The lung sections notably do not resemble inflamed lungs with severe infiltration and alveolar thickening that has been observed in previous studies in mice receiving LPS [62]. Accordingly, while the Al-based MOFs and alum do have powerful immunogenicity, they do not cause gross airway inflammation at this early time point as revealed via histological analysis.

### Al-based MOFs remain localized to the lungs following instillation and have minimal accumulation

**Fig. 9:**
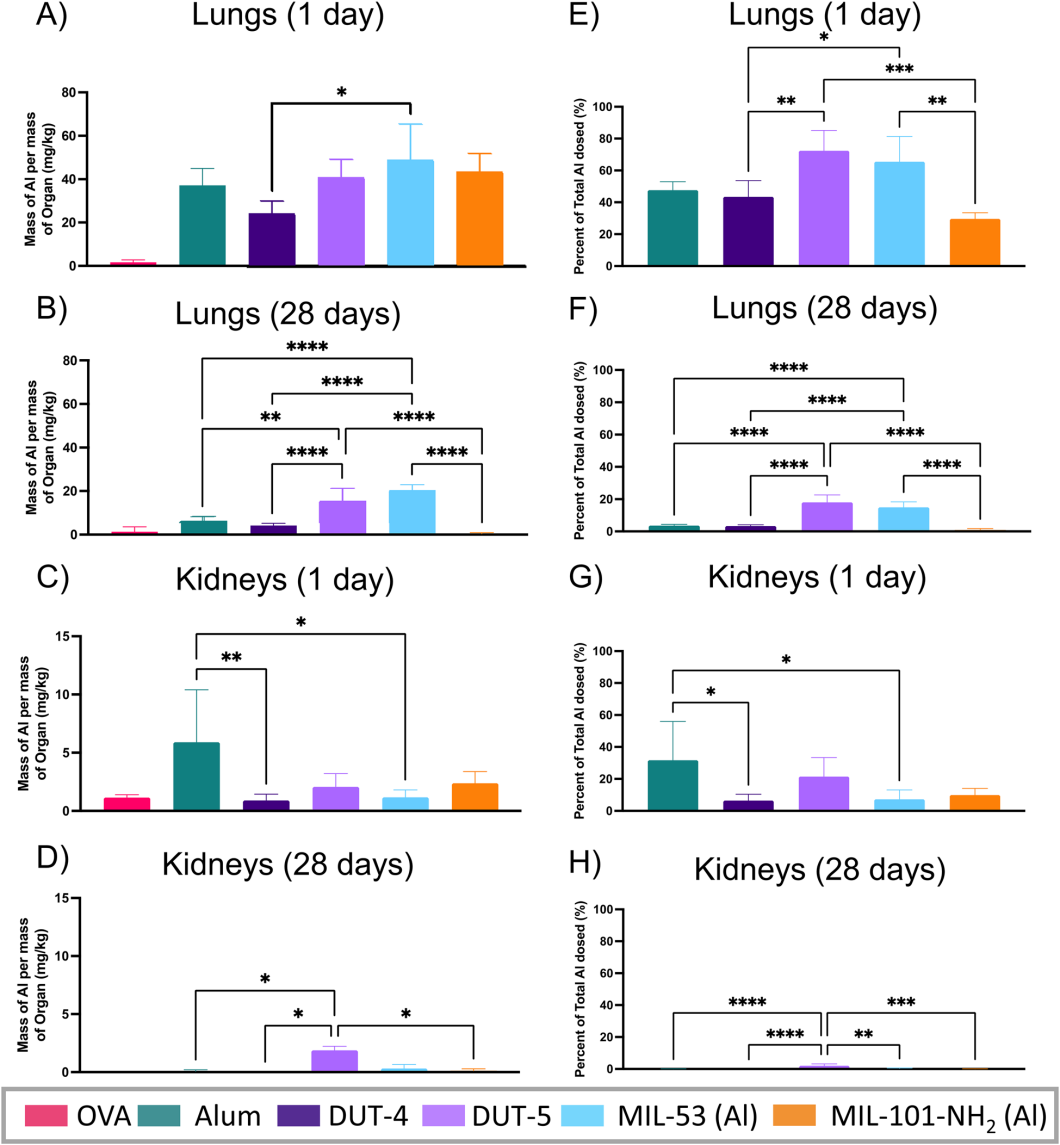
Al murine biodistribution following at acute and vaccine dosages. Biodistribution of different Al-based NPs measured as mass of Al per mass of organ (mg/kg) at **A** 24 h after initial dosage in the lungs, **B** 28 days after initial dosage in the lungs, **C** 24 h after initial dosage in the kidneys, and **D** 28 days after initial dosage in the kidneys. Biodistribution results from **A**–**D** are reported as a percentage of mass of Al dosed in **E**–**H**. Statistics shown are multiple comparisons via Tukey’s multiple comparison tests as part of a one-way ANOVA (excluding the OVA control; *p < 0.05, **p < 0.01, ***p < 0.001, ****p < 0.0001). Comparisons not shown indicate that no statistically significant difference. (*N* = 4 for alum and OVA, *N* = 5 for all Al-based MOFs). All data show the mean and SD

Finally, to better understand the localization and clearance of the dosed Al-based NPs, the distribution of NPs in major organs was determined for the different treatment groups at 24 h after the prime dosage and at day 28, 14 days after receiving two immunization dosages (amounts, times, dosage, and N also summarized in Table 1). The distribution of NPs at the two respective time points was determined via organ digestion followed by ICP-MS analysis to detect Al content. The results of this biodistribution study, which shows the mass of Al from the respective particles in different organs as well as the percentage of Al expected from dosage, for the lungs and kidneys are shown in Fig. 9. Other organs tested include the heart, liver, spleen, and blood of the mice consistently showed background levels of aluminum both at 24-h and 28-day time points for all treatments (Additional file 1: Figs. S13 and S14).

As shown in Fig. 9a, c, the vast majority of aluminum for the Al-based NPs remained in the lungs at the 24-h time point, as expected given the orotracheal administration of the Al-based NPs. This result indicates generally effective localization to lungs, as desired. This localization is also reflected in Fig. 9e, which shows the percentage of total Al dosed remaining in the lungs after 24 h for each of the respective Al-based NPs. This percentage was calculated using the mass of Al in a single dose for each of the treatments, which was also determined via ICP-MS (Additional file 1: Table S1). Together, Fig. 9a, e demonstrate a surprising result, that less than 50% of the Al from the alum, DUT-4, and MIL-101-NH_2_ (Al) remain in the lungs 24 h after dosage while 65% and 73% of the Al from MIL-53 (Al) and DUT-5 remain in the lungs. This result is unexpected given the size of the NPs, particularly alum and MIL-101-NH_2_ (Al), which have particle sizes around 1 µm. The stability of these MOFs in the lung may account for this result. Previous work has noted that MIL-101-NH_2_ (Fe) and DUT-4 are unstable in water and show reduced crystallinity over the course of a few days’ time; however, these works also cite DUT-5 among the water-unstable MOFs, which did not demonstrate this rapid mass loss from the lungs [45, 63]. This result indicates that the water stability of the MOFs, which likely have similar breakdown mechanisms vie protonation of their carboxylate ligands, is likely not responsible for their low retention in the lungs at 24 h [62]. This lower retention may be partially explained by the presence of Al-based NPs in the kidneys (Fig. 9c, g), particularly of alum, which would suggest MOF clearance from the lung proceeds through renal excretion. However, the mass of Al in the kidneys does not account for nearly all of the mass loss within the first 24 h. It is possible that the clearance of some of the Al-based NPs is more rapid than others potentially because of enzymatic breakdown in the airspace and/or intracellular degradation and may warrant investigation of distribution at pre-24-h time points as well as detection of Al in urine and feces of the mice for a complete mass balance [64]. Another possibility is mass losses from the instillation which could lead to delivery to other organs, although this is thought to be minor [65].

The overall rapid NP clearance from the lung proceeds as expected, leading to significantly reduced amounts of Al in the lungs at the 28-day time point (shown in Fig. 9b), despite a second dosage of Al-based NPs at the 14-day time point. This vastly reduced Al content is further reflected in Fig. 9f, which shows that at most, only 18% of the dosed Al remains in the lungs at this time point (DUT-5, which would be expected to be fully cleared by around 40-days based on a half-life estimation from our limited lung timepoints, Additional file 1: Table S3). Unlike at the 24 h time point, there is no clear indication of clearance through the kidneys at this time point (Fig. 9d, h), though this is still possible to be the route of clearance throughout the 14 days prior with little Al remaining at this point after excretion by the mice [66]. The potentially more rapid clearance of alum, DUT-4, and MIL-101-NH_2_ (Al) noted at the 24 h time point is further demonstrated in the 28-day biodistribution data as well for which alum only has 3.5% of its total mass of Al remaining in the lungs (Fig. 9e), while the DUT-5 and MIL-53 have 18%, and 15% of the total Al mass remaining in the lungs. The MOFs with similar clearance rates are DUT-4 and MIL-101-NH_2_ (Al), which were the sample NPs with lower percentages of Al remaining in the lungs at the 24 h time point. Furthermore, the more rapid clearance of the alum, DUT-4, and MIL-101-NH_2_ (Al) may be related to their less effective generation of IgA titers (Fig. 5a), especially relative to DUT-5, which also has the most effective activation of APCs in vivo (Fig. 7). The clearance may lead to reduced depot formation, one of alum’s mechanisms of action [13]. Accordingly, it is possible that DUT-5’s longevity is advantageous for its IgA antibody generation; however, this mechanism cannot solely account for its greater performance because MIL-53 (Al)’s similar longevity did not lead to comparable IgA antibody titers. Thus, it is possible that airway retention coupled with APC activation may account for DUT-5’s greater mucosal antibody generation, which may be related to both the number and size of NPs (Table 2) as well as the aluminum content per particle.

## Conclusions

This work serves as the first to explore the use of Al-MOF NPs as pulmonary vaccine adjuvants and adjuvants more broadly. The Al-based MOFs have demonstrated a number of advantages toward vaccine applications including APC activation and even greater antibody titers in the serum (IgG) and the lungs (IgA) than the well-studied and widely used adjuvant, alum. Furthermore, DUT-5-vaccination has demonstrated generation of IgG2a antibodies, which indicates generation of a low level cellular response in addition to the humoral response common for alum. Furthermore, the Al-MOFs have aerodynamic sizes more ideal for alveolar deposition than alum with DUT-4, DUT-5, and MIL-101-NH_2_ (Al) all having MMADs within the 1.5–2.5-µm size range, while alum’s greater MMAD of a ~ 4 µm corresponds with an estimated ~ 20% lower projected airway deposition. In particular, DUT-5 tended to perform best (in some cases equivalent to other treatments) in all of the aforementioned metrics and also outperformed the alum by more than an order of magnitude in terms of IgA and IgG antibody generation when mice were vaccinated at equivalent amounts of aluminum with alum and DUT-5 (alongside OVA).

This study provides a critical starting point for the further exploration and use of MOFs as pulmonary vaccine adjuvants; however, additional studies expanding the number of mice per study to increase statistical power, dosing particles in vitro alongside antigen to explore potential synergy for APC activation, and controlling of parameters such as particle size and particle number, which were not controlled for here, would further enhance the characterization of the effects of the unique features of each MOF. For example, modulating the synthesis of the MOFs to yield NPs of constant particle size, potentially via modulation of water content, would allow for direct comparison of results as a function of particle chemistry or Al content [67]. Further studies could also be performed to isolate the effects of particular metal clusters by synthesizing Al-MOFs with identical organic linkers. Additionally, more complete safety profiles of the MOFs need to be implemented to fully evaluate their potential as pulmonary vaccine adjuvants including more complete clearance timelines of the Al-MOFs, histological assessment following repeated dosing, and better understanding of factors such as IC50 to more completely evaluate their in vivo safety. Kinetic histological studies and cytokine secretion profiles could aid in this goal. Better understanding of MOF trafficking would be beneficial which could be accomplished via addition of several timepoints over the course of the first 48 h to complete the mass balance for Al-based MOF delivery. Lymph node trafficking and education of antigen-specific T cells would further make these studies more robust, as could alternative delivery routes and challenge models to explore MOF vaccine applications and effectiveness. Finally, our work demonstrates adjuvant vaccination with the model antigen OVA; this work sets the stage for future studies administering Al-MOFs with relevant subunits antigens, especially with those to protect against airway-specific pathogens, and appropriate challenge models to test the overall efficacy.

Overall, this work serves as a critical step toward yielding effective adjuvants for pulmonary vaccination. The particles explored are porous, tunable NPs of ideal aerodynamic size for delivery to the alveolar region of the lungs that generate strong antibody responses both locally and systemically, in many cases even more so than alum, a proven adjuvant. These findings represent important proof-of-concept demonstrations of the benefit of Al-based MOF NPs as stimulatory adjuvants and potential vehicles for pulmonary vaccination.

## Supplementary Information

**Additional file 1.** Additional supplemental methods, figures, and calculations can be found in the associated file.

## Data Availability

The datasets generated during and/or analyzed during the current study are available from the corresponding author on reasonable request.
