# Supplementary material for: Aluminum-based metal–organic framework nanoparticles as pulmonary vaccine adjuvants
Source: J Nanobiotechnology. 2023 Feb 3;21:39. doi: 10.1186/s12951-023-01782-w (PMC9896814; doi:10.1186/s12951-023-01782-w)
Supplement: Supplementary file 1 — Additional file 1. Additional supplemental methods, figures, and calculations can be found in the associated file. [file 12951_2023_1782_MOESM1_ESM.pdf]

Supporting Information for:

Aluminum-Based Metal-Organic Framework Nanoparticles as Pulmonary Vaccine Adjuvants

Zachary S. Stillman,[1] Gerald E. Decker,[2] Michael R. Dworzak,[2] Eric D. Bloch,[2] Catherine, A. Fromen[1,*]

[1]Department of Chemical and Biomolecular Engineering

[2]Department of Chemistry and Biochemistry

University of Delaware, Newark, DE 19716

*corresponding author.
cfromen@udel.edu
150 Academy St.
Newark, DE 19716
(302) 831-3649

Table of Contents

Figures S1-S4: Powder X-Ray Diffraction (PXRD) patterns 3
Figures S5-S7: Gas Adsorption Measurements 4
Figure S8: Viability of Al-based NPs by RAW264.7 cells. 5
Figure S9: Flow cytometry gating for NP uptake by RAW 264.7 cells. 6
Figure S10: Uptake images for RAW264.7 cells........ 6
Figure S11: Antibody titers from equivalent Al Dosage Study. 7
Figure S12: Additional Histology Images 8
Figure S13: Biodistribution mass measurements........ 9
Figure S14: Biodistribution %mass measurements 10
Table S1: Particle characteristics of Al-based NPs........ 11
Table S2. Particle Number Approximation 11
Table S3. NP Clearance Approximation........ 12

Figures S1-S4: Powder X-Ray Diffraction (PXRD) patterns

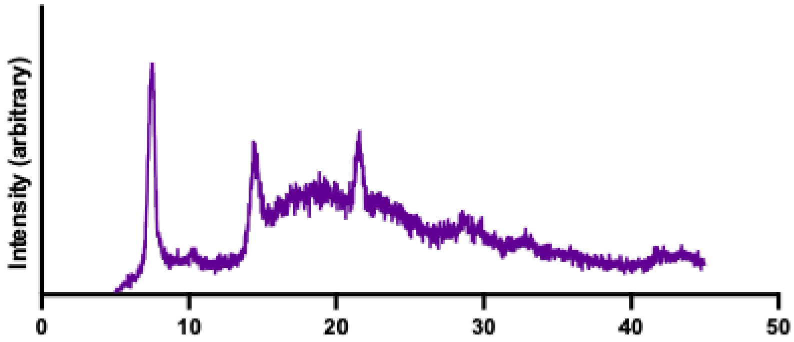

Figure S1: Powder X-Ray Diffraction (PXRD) pattern for DUT-4 NPs.

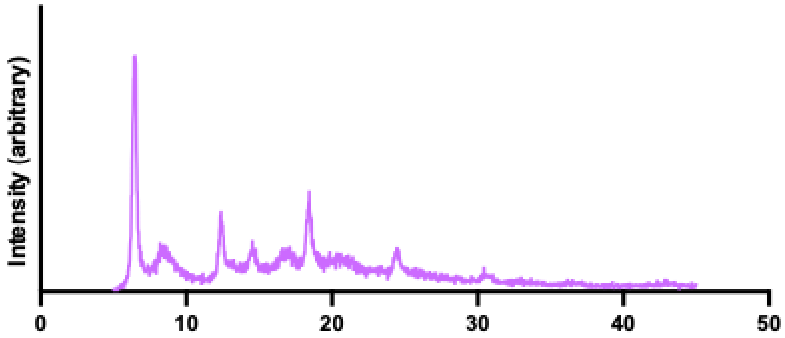

Figure S2: Powder X-Ray Diffraction (PXRD) pattern for DUT-5 NPs.

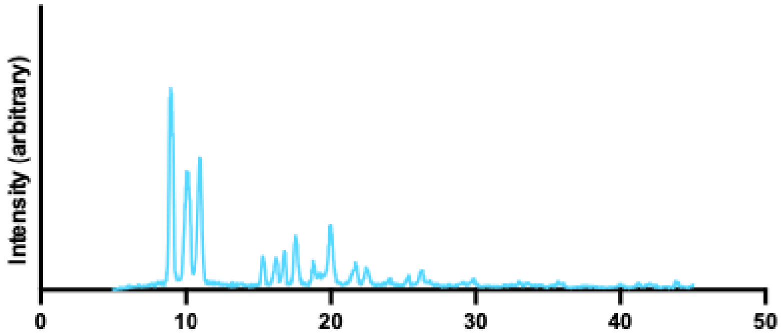

Figure S3: Powder X-Ray Diffraction (PXRD) pattern for MIL-53 (Al) NPs.

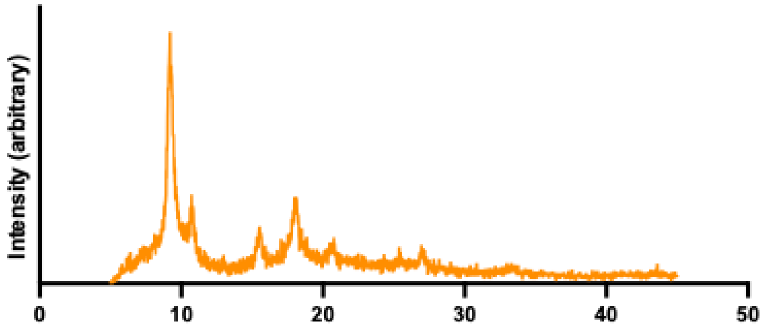

Figure S4: Powder X-Ray Diffraction (PXRD) pattern for MIL-101-NH_2 (Al) NPs.

Figures S5-S7: Gas Adsorption Measurements

Following washing in solvent, the respective powders were dried under nitrogen. Low-pressure gas adsorption isotherms were measured on a Micromeritics Tristar II PLUS at 77 K. The respective NPs were loaded into a gas adsorption tube and degassed overnight at 100 °C under flowing nitrogen. Upon activation the sample was analyzed with nitrogen at 77 K.

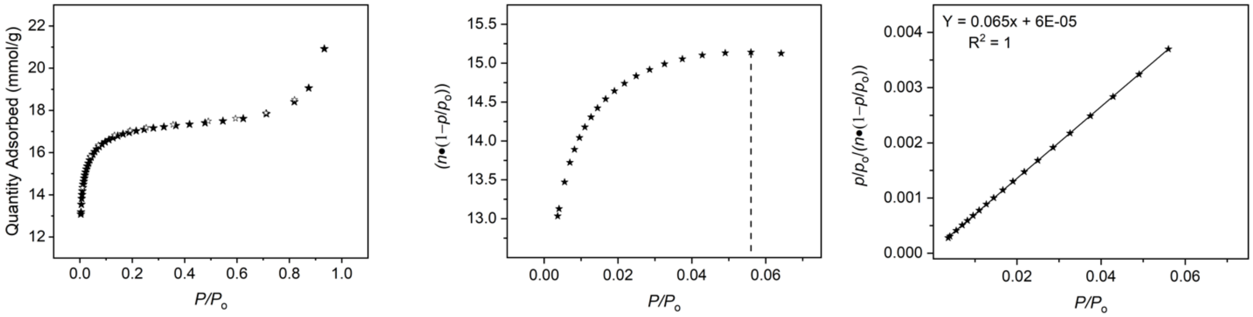

Figure S5: Left: N_2 adsorption (solid black stars) and desorption (hollow black stars) at 77 K for DUT-4. Middle: Plot of n(1-P/P_0) vs. P/P_0 to determine the maximum P/P_0 used in the BET linear fit according to the first BET consistency criterion for N_2 adsorption at 77 K. Right: The slope of the best fit line for $P/P_0 < 0.0560$ is 0.065 and the y-intercept is 0.00006, which satisfies the second BET consistency criterion. This results in a measured surface area of 1501 m^2/g to N_2.

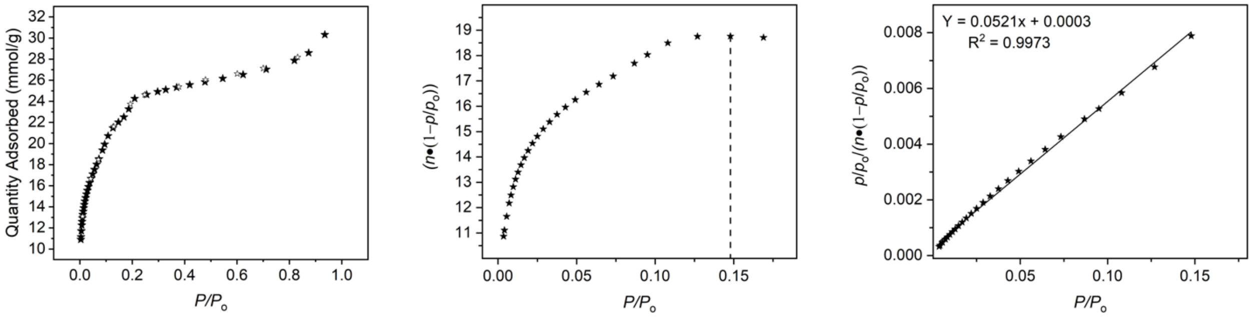

Figure S6. Left: N_2 adsorption (solid black stars) and desorption (hollow black stars) at 77 K for MIL-53 (Al). Middle: Plot of n(1-P/P_0) vs. P/P_0 to determine the maximum P/P_0 used in the BET linear fit according to the first BET consistency criterion for N_2 adsorption at 77 K. Right: The slope of the best fit line for $P/P_0 < 0.0272$ is 0.0764 and the y-intercept is 0.00002, which satisfies the second BET consistency criterion. This results in a measured surface area of 1277 m^2/g to N_2.

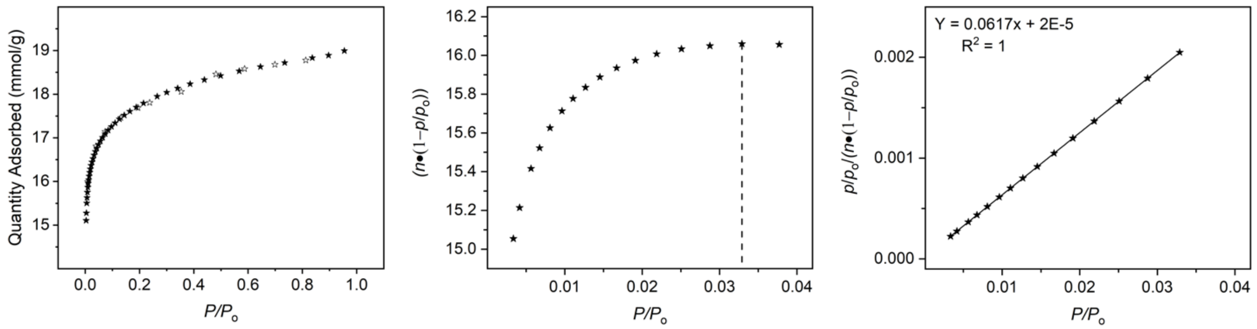

Figure S7: Left: N_2 adsorption (solid black stars) and desorption (hollow black stars) at 77 K for MIL-101-NH_2 (Al). Middle: Plot of n(1-P/P_0) vs. P/P_0 to determine the maximum P/P_0 used in the BET linear fit according to the first BET consistency criterion for N2 adsorption at 77 K. Right: The slope of the best fit line for P/P_0 < 0.1479 is 0.0521 and the y-intercept is 0.0003, which satisfies the second BET consistency criterion. This results in a measured surface area of 1873 m^2/g to N_2.

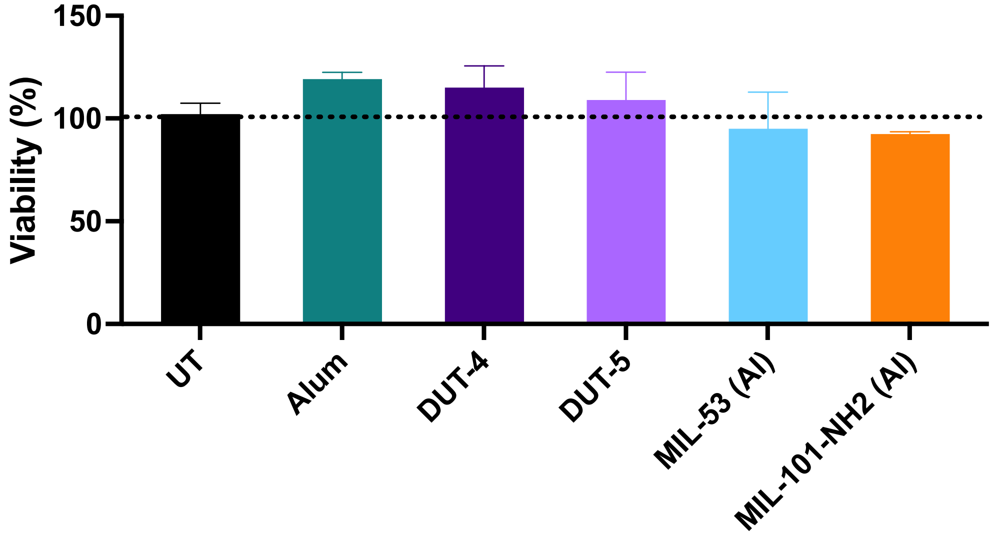

Figure S8: Viability of Al-based NPs by RAW264.7 cells.
Viability of Al-based NPs by RAW264.7 cells 24 h after dosage with Al-based NPs at 10 µg/mL (N = 3). Data shows means with error bars for SD.

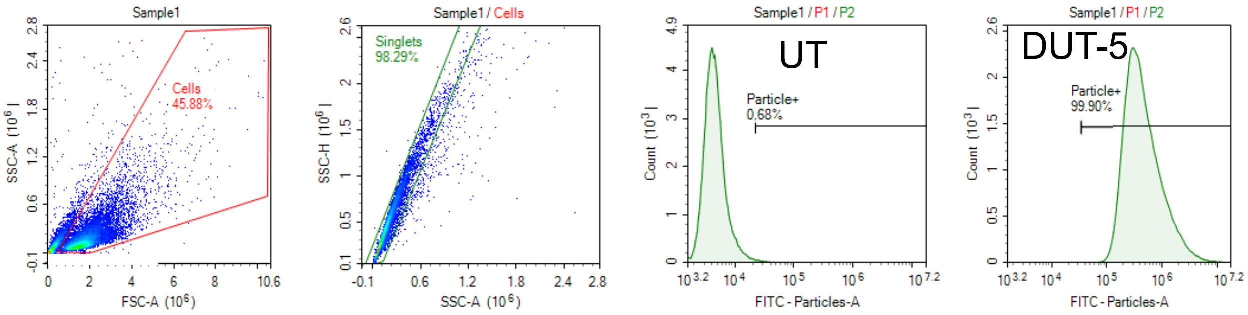

Figure S9: Flow cytometry gating for NP uptake by RAW 264.7 cells.
Cells were dosed at 100 µg/mL of particles loaded with FITC and uptake determined via the gating scheme shown above for untreated (UT) with an example of uptake for DUT-5-dosed cells.

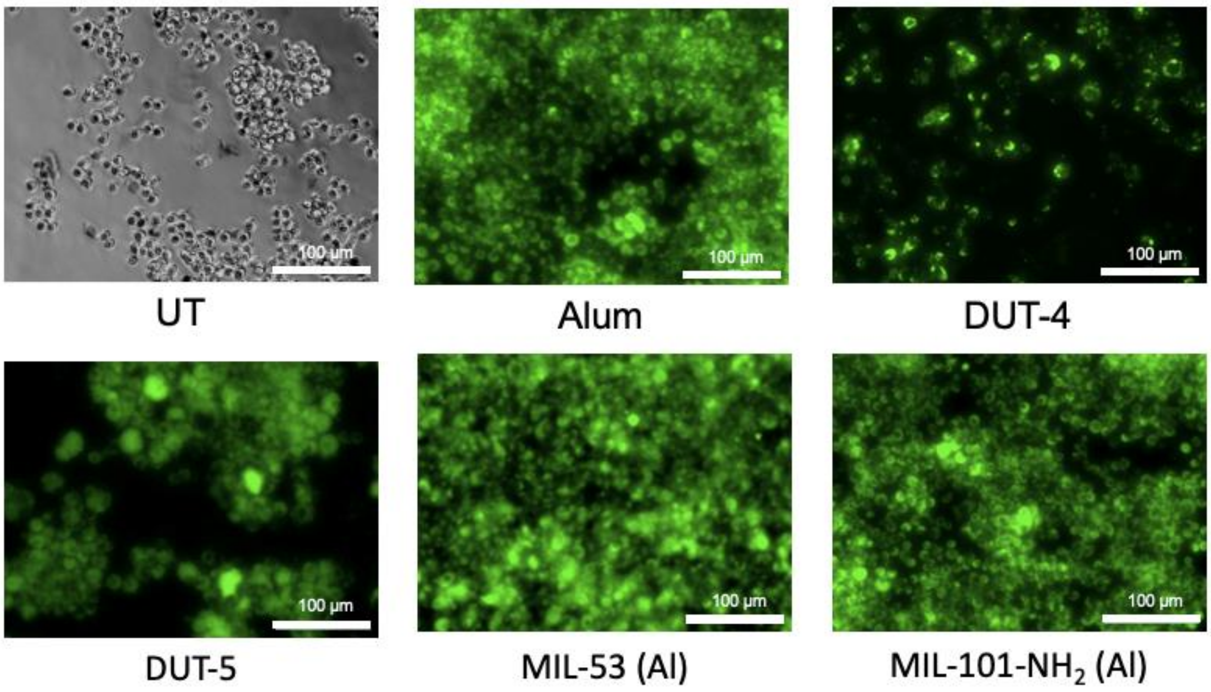

Figure S10: Uptake images for RAW264.7 cells.
Image labeled "UT" shows brightfield image of untreated RAW cells, while the other images indicate the label of the Al-MOF with which they were dosed and show only the GFP channel (in which FITC will show up) to demonstrate the ubiquity of uptake in addition to the images in the main text Figure 3.

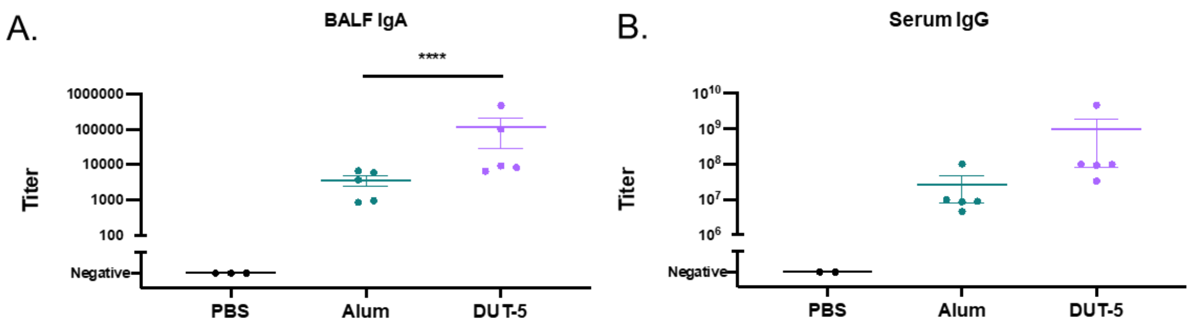

Figure S11: Antibody titers from equivalent Al Dosage Study.
A) Bronchoalveolar lavage fluid (BALF) IgA and B) serum IgG antibody titers from equivalent Al dosage study

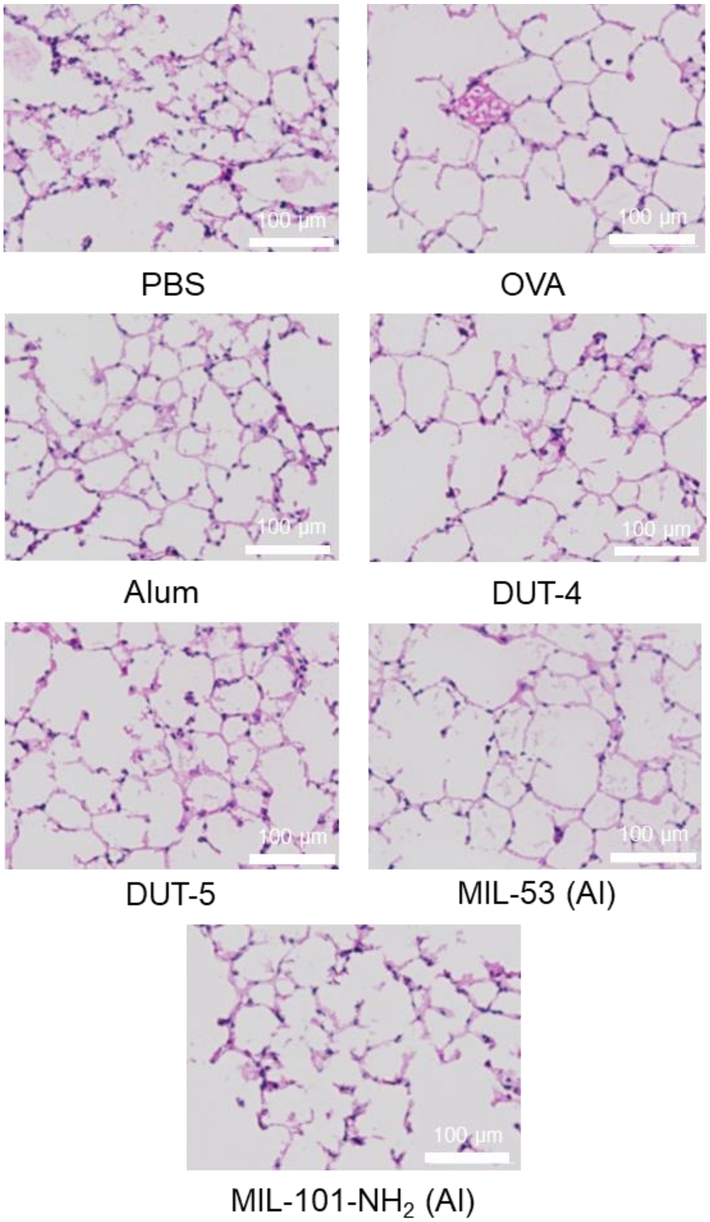

Figure S12: Additional Histology Images
Histological analysis of lungs from mice dosed with PBS, OVA, alum, and the four respective Al-based MOFs, DUT-4, DUT-5, MIL-53 (Al), and MIL-101-NH_2 (Al). The images were taken of the H&E-stained lung sections and all have equivalent scale bars representing 100 µm.

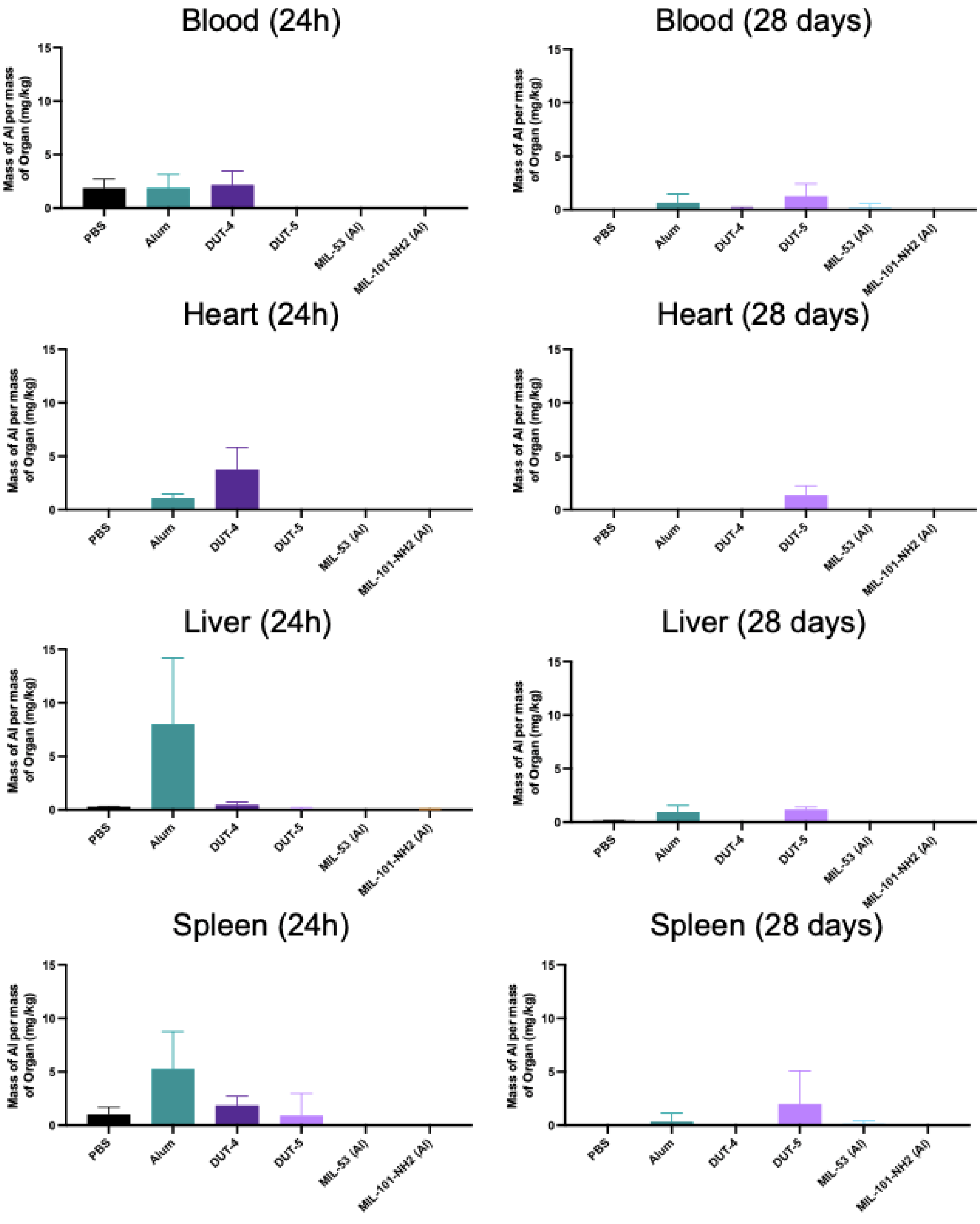

Figure S13: Biodistribution mass measurements

Biodistribution of different Al-based NPs measured as mass of Al per mass of organ (mg/kg) at 24 h after initial dosage and 28 days after initial dosage (including booster dose) in various organs/sources. (N=2 for PBS, N=4 for alum, N=5 for all Al-based MOFs). All data show the mean and SD.

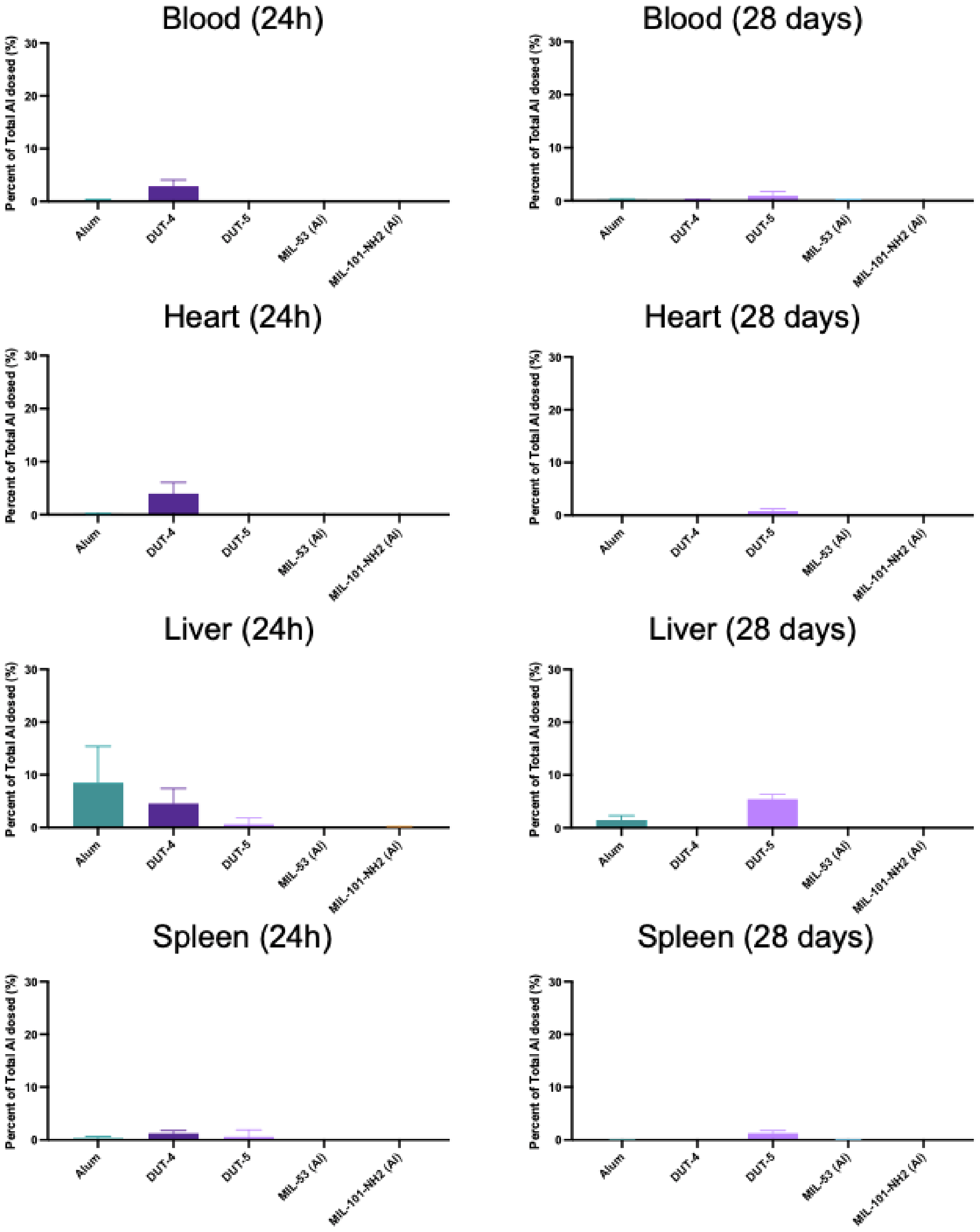

Figure S14: Biodistribution %mass measurements
Biodistribution of different Al-based NPs measured as mass% of total Al dosed at 24 h after initial dosage and 28 days after initial dosage (including booster dose) in various organs/sources. (N=4 for alum, N=5 for all Al-based MOFs). All data show the mean and SD.

Table S1: Particle characteristics of Al-based NPs.
The table below notes the measured hydrodynamic diameters, geometric diameters, and zeta potentials for each of the Al-based NPs determined from DLS, SEM, and zeta potential measurements, respectively (N=3).

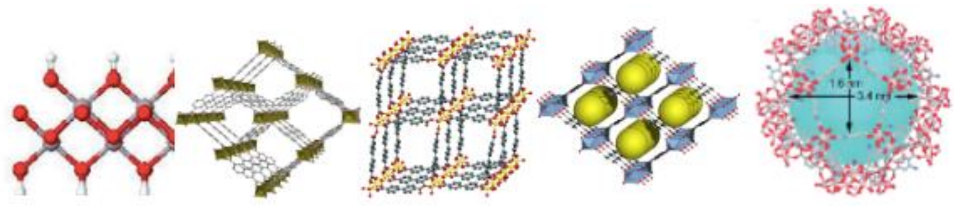

	Alum	DUT-4	DUT-5	MIL-53 (Al)	MIL-101 NH_2 (Al)
Hydrodynamic Diameter (nm)	1934 ± 660	205 ± 1	479 ± 10	1354 ± 35	560 ± 5
Geometric Diameter (nm)	513 ± 127*	209 ± 38	465 ± 96	1621 ± 430	374 ± 119
Zeta Potential (mV)	-32.6 ± 1.9	-9.6 ± 0.2	-21.5 ± 1.3	-29.0 ± 1.4	-21.5 ± 0.8

*Measurements of discernable individual particles. Larger aggregates were also present.

Table S2. Particle Number Approximation

Calculation formula is noted below. Prior to calculation, the parameters of "volume per particle" and "molar mass per volume" were approximated for each of the respective particles.

"Volume per particle" was approximated using the SEM images taken to determine particle dimensions, which were subsequently converted into approximate average volume for 1 particle.

"Molar mass per volume" was approximated using the structures of the respective NPs alongside dimensions of the metal clusters/ligands for the Al-based MOFs and the Al(OOH) structure for alum. With these values, a calculation of approximate molar mass per volume based on the structures were performed for each respective NP.

These values were then plugged into the formula below alongside the known masses dosed in each of the respective *in vivo* studies, leading to the approximated values reported in **Table 2**.

***Equation 1*: ** Formula used to approximate the number of particles per dosage for *in vivo* studies. Units are noted [in brackets], as are the units for the formula following the [=] symbol.

$$Approx.\ Number\ \left[\frac{particles}{dosage}\right] = \frac{Mass\ of\ particles}{dosage} \times \left(\frac{Volume}{1\ particle}\right)^{-1} \times \left(\frac{Mass\ per\ mole}{Volume}\right)^{-1} \times N_A$$

$$[=]\frac{mass}{dosage} \times \left(\frac{particles}{volume}\right) \times \left(volume \times \frac{moles}{mass}\right) \times \frac{6.022E23\ particles}{mole}$$

Table S3. NP Clearance Approximation

Utilizing the biodistribution data from days 1 and 28, clearance half-lives from the lungs were approximated for each of the four respective Al-MOFs as well as alum, fitting to the form of the following equation:

$$N(t) = N_0 \left(\frac{1}{2}\right)^{\frac{t}{t_{1/2}}}$$

where $N(t)$ is the amount of material remaining at time t, N_0 is the initial amount of material, and $t_{1/2}$ is the half-life of the material. Equation S1 could be fit using the "percent of expected remaining" data converted to fraction remaining as $\frac{N(t)}{N_0}$ with day 0 as a value of 1 and an increase of 1 at day 14 for the booster dose.

Using these half-lives, the expected clearance times for the Al-MOFs were determined, particularly for DUT-5 and MIL-53 (Al), which were not fully cleared by day 28. Utilizing the uncertainty of the approximated half-lives, lower end clearance times were also estimated. Alum and DUT-4 do not have expected lower end clearance times as their full clearance occurs at approximately the 28-day time point.

Al-based NP	Approximate Clearance Half Life (days)	Expected Clearance Time (days)	Lower End Clearance Time (days)
**Alum**	2.90	28.2	N/A
**DUT-4**	2.83	27.9	N/A
**DUT-5**	5.23	40.5	31.5
**MIL-53 (Al)**	4.79	38.1	29.2
**MIL-101-NH_2 (Al)**	2.09	24.1	16.8
